# Inconsistently reporting post-licensure EPA specifications in different clinical professions hampers fidelity and practice translation: a scoping review

**DOI:** 10.1186/s12909-023-04364-4

**Published:** 2023-05-24

**Authors:** Sonya J. Moore, Thorlene Egerton, Mark Merolli, Jessica Lees, Nino La Scala, Selina M. Parry

**Affiliations:** 1 Department of Physiotherapy, School of Health Sciences, The University of Melbourne, Level 7, Alan Gilbert Building, Victoria, 3010 Australia; 2 Faculty of Health and Centre for Research in Assessment and Digital Learning, Deakin University, Deakin, Australia

**Keywords:** Entrustable professional activities, Clinical education, Postgraduate, Medicine, Interprofessional

## Abstract

**Background:**

Entrustable Professional Activities (EPAs) are defined units of professional practice entrusted to professionals once they have attained the specific competencies required to complete the end-to-end task. They provide a contemporary framework for capturing real-world clinical skillsets and integrating clinical education with practice. Our scoping review question was: how are post-licensure EPAs reported in peer reviewed literature, in different clinical professions?

**Method:**

We followed the Preferred Reporting Items for Systematic Reviews and Meta-Analyses Extension for Scoping Reviews (PRISMA-ScR) checklist, Arksey and O’Malley and Joanna Briggs Institute (JBI) methodology. Searching ten electronic databases returned 1622 articles, with 173 articles included. Data extracted included demographics, EPA discipline, titles and further specifications.

**Results:**

All articles were published between 2007–2021 across sixteen country contexts. The majority were from North America (*n* = 162, 73%) describing medical sub-specialty EPAs (*n* = 126, 94%). There were comparably few EPA frameworks reported in clinical professions other than medicine (*n* = 11, 6%). Many articles reported only EPA titles without further explanation and limited content validation. The majority did not include information about the EPA design process. Few EPAs and frameworks were reported according to all the recommended EPA attributes. There was unclear distinction between specialty-specific EPAs and those that could be useful across disciplines.

**Discussion:**

Our review highlights the large volume of EPAs reported in post-licensure medicine, including the volume disparity compared to other clinical professions. Basing our enquiry upon existing guidelines for EPA attributes and features, our experience in conducting the review and our primary finding demonstrated heterogeneity of EPA reporting according to these specifications. To promote EPA fidelity, and quality appraisal, and to reduce interpretation subjectivity, we advocate: diligently reporting EPA attributes and features; including reference or citation to EPA design and content validity information; and considering distinguishing EPAs as specialty-specific or transdisciplinary.

**Conclusion:**

A large volume of post-licensure EPAs were identified in medicine relative to other clinical professions. EPA specifications were absent or variously reported in the literature, risking ambiguous interpretation. The authors recommend that future EPAs are reported with reference to established and evolving construct recommendations, which is integral to concept fidelity and translation to practice and education.

**Supplementary Information:**

The online version contains supplementary material available at 10.1186/s12909-023-04364-4.

## Background

Clinical skills can be characterised as the practical and interpersonal skills required by health professionals to engage in and deliver a clinical healthcare service. Clinical skills may contain examination, practical procedures, communication and management skills with components of "how to", why, and the reasoning of what the outcomes might mean [1]. Individual clinical competencies refer to the performance of a specific learnable skill, often contextualized within a more broadly designated role or domain [2]. Benchmarking of individual clinical competencies is well described for pre-licensure entry-to-practice health professions including dentistry, medicine, paramedicine, pharmacy, physiotherapy, podiatry and psychology [3] and post-entry-to-residency medical specialties [4].

Entrustable Professional Activities (EPAs) are well-defined units of professional practice that can be entrusted to a professional once they have attained and can contextually link the competencies required to complete the end-to-end task [5]. They offer a framework for observing and demonstrating the translation of competencies into real-world clinical practice or work units [6, 7]. They are increasingly recognized as better representing contextualized performance of clinical skills in practice than assessing piecemeal clinical skills or granular competencies [8].

Several recent investigations and scoping reviews have described the use of EPAs in entry-to-practice health professional education [9–13]. These EPAs aim to capture and assess the clinical skills required for proficient, independent clinical practice upon initial licensure and entry-to-practice as a health professional, with key intentions to align clinical skill performance with expectations and learner assessment [9]. These EPAs represent entry-to-practice clinical skills, typically acquired in pre-licensure education.

Differentially, post-licensure clinical practice as a qualified clinician requires development and assessment of increasingly complex and specialist clinical skills. These skills are typically developed in post-licensure professional education. This includes managing unexpected challenges, higher order thinking, navigating uncertainty and making decisions in challenging situations at the “trusted” apex of Miller’s pyramid [14–16]. Post-licensure professional education and practice can be differentiated (from entry-to-practice level) by the advancing integration of a complex body of knowledge, including specialized knowledge *and* independent critical analysis, with complex information synthesis aligned to originality of circumstances and challenges [14].

A systematic review by O’Dowd and colleagues [17] and an updated scoping review by Liu and colleagues [18] synthesized work on the use of EPAs in postgraduate medicine. Our review sought to extend the context and focus on EPAs in different clinical professions, including any subsequent publications. By definition EPAs are discipline-specific and the viability of collaborative interprofessional EPAs is contentious [19], although the concept and value of transdisciplinary EPAs has been recently recognised [20]. Further, some authors have questioned the validity of EPAs, particularly where they are insufficiently designed or described according to the ten Cate et al. [21] criteria[22]. This review does not examine or challenge these perspectives, rather it explores the landscape of how existing EPAs are reported in the context of different clinical professions including and beyond medicine. This is relevant to inform discipline-specific education, interprofessional health education and opportunities to progress the field of post-licensure and transdisciplinary EPAs.

## Method

### Identifying the research question

We took a broad view to answer our research question: how are post-licensure EPAs reported in peer reviewed literature, in different clinical professions? Understanding the current perspective and landscape of EPAs representing post-licensure clinical practice is necessary to advance the future direction of post-licensure professional clinical education and practice frameworks. This scoping review extends on the current perspective of how EPAs are used in (i) pre-licensure healthcare and (ii) medicine only.

To address recognized conventions, nuance and diversity of common clinical and education language, we defined the following terms of reference central to our enquiry:

- EPA: A well-defined and conceptualized work unit of professional practice which requires contextualized integration of multiple competencies to proficiently complete [5].
- Clinical competency: A defined granular ability or specific learnable skill required for safe and proficient clinical practice matched to the context [5, 8, 15].
- Post-licensure professional clinical practice: Representing the common-language term of reference for practice as a qualified clinician, *after* completion of the initial entry-to-practice qualification. (The timing of unrestricted medical licensure is not standardised nationally or internationally [23]. The CanMEDS framework groups seven roles of competent physicians and provides an internationally relevant model of practice for all clinical health professions [24]. Eligibility for medical licensure begins at the CanMEDS stage of “Entry to Residency” [25]. We therefore defined post-licensure professional practice as that which follows an entry-to practice medical degree, with residency at the starting point.)
- Discipline-specific clinical skills: Technical, procedural and/or cognitive skills which are defined by the discipline-specific complex body of knowledge, including specialist knowledge.
- Interpersonal clinical roles: These are reflective of CanMEDS intrinsic roles [25] and include thought patterns, behaviours and growth mindset that transcend disciplines to define a good clinician [20, 26].

A preliminary search of the Cochrane Library, JBI Evidence Synthesis, MEDLINE and Open Science Framework did not identify any current or underway systematic or scoping reviews relating to post-licensure EPAs. Our preliminary search indicated a current perspective of existing EPA recommendations with terminology nuances and piecemeal application, therefore scoping review methodology was appropriate to discover and appraise the overall body of available literature. Guided by Thomas and colleagues’ recommended approach [27] we synthesized and represented the literature numerically and thematically. We adopted the subjectivist epistemological perspective advocated by Thomas and colleagues [28].

We followed the Preferred Reporting Items for Systematic Reviews and Meta-Analyses Extension for Scoping Reviews (PRISMA-ScR) checklist [29], Arksey and O’Malley [30] and Joanna Briggs Institute (JBI) methodology [31] with additional refinements aligned with Levac and colleagues [32]. We prospectively registered the final protocol with Open Science Framework Registries on November 24, 2021 [33].

### Identifying relevant articles

Detailed inclusion and exclusion criteria for our review are contained in Table 1. Included articles reported EPAs in the context of post-licensure clinical practice and involve direct human patient interaction. We included all article types. Articles relating to entry-to-practice, pre-licensure or not entailing direct patient interaction were excluded. Unlike previous reviews [17, 18], we did not narrow our inclusion to medicine or non-medicine disciplines.

**Table 1:** Inclusion and exclusion criteria. This table contains the article inclusion and exclusion criteria for this review. Abbreviations: EPA – Entrustable Professional Activities

Characteristics	Inclusion	Exclusion
**Article type / study design**	Systematic reviews Scoping reviews Primary research studies Delphi studies Reviews of existing EPAs	Conference abstracts Blogs Letters to editor Reviews that do not contain an EPA
**Context**	Clinical healthcare professions providing patient consultations in a post-professional context (post-licensure practice as a qualified clinician) Includes but not limited to the following post-professional clinical disciplines: medicine, medical resident / registrar, medical fellowships, medical specialties beyond the foundation medical qualification, physiotherapy, pharmacy, psychology, nursing, dentistry	Healthcare contexts without direct patient interaction eg. health administration Clinical healthcare context relating to pre-qualification Clinical healthcare or education during or relating to the entry-to practice stage
**Concept**	Any clinical healthcare EPA with an advanced practice context Includes information sources describing but not limited to EPA: Design/construct, application, efficacy, critique, research and development	EPAs relating to pre-qualification or entry-to-practice stages Fragmented skills or competencies which are not contextualised within performance of a whole task Administrative/regulatory healthcare process Laboratory procedures
**Publication**	No publication or language restrictions applied on the initial search	Non-English language studies were excluded from further review at the full text stage

### Information sources and selection of evidence

Our research team and author panel were constructed to contribute scholarship and experience across interprofessional research, clinical education and clinical practice to generate varied relevant perspectives [34] to our enquiry, interpretation and recommendations. Our systematic search strategy was developed in consultation with a senior librarian (VB) (See Supplementary Table 1 – MEDLINE search strategy). Eight databases were searched from inception on September 16 2021 without restrictions to language or publication status: Ovid MEDLINE, CINAHL (EBSCOhost) Embase (OVID); SPORTDiscus; PsychInfo, (Education Research Complete (ERIC), Evidence Based Medicine Reviews (EBMR) (including Cochrane) and Joanna Briggs Institute’s Database of Systematic Reviews and Implementation Reports. Two gray literature databases MedNar and OpenGray were subsequently searched from inception to September 21 2021. Reference lists of included articles were hand-searched for additional peer review literature sources.

All articles were imported and managed in Covidence [35]. Two of five independent reviewers (SM, TE, NLS, MM, JL) reviewed articles at each of the two stages of study selection against the predefined criteria (see Table 1 – Inclusion and exclusion criteria). Firstly, titles/abstract were screened with all conflicts included in the next stage of full text review, where a more accurate assessment of concept and context was undertaken. Secondly, at the full text review stage we encountered great variety in how articles defined or described EPAs, presenting challenges in discerning the reported concept and/or context of the EPA. Any uncertainty or discrepancies between reviewers were resolved through discussion and consensus between the two conflicted reviewers.

### Data charting process and data items

A data charting form was prospectively developed and piloted to capture study design, country, discipline context, EPAs attributes, any further specifications and alignment to professional competency standards (see Supplementary file – Data charting template). Data were charted in Covidence [35] exported to Microsoft Excel (2016) and cross checked. All reviewers iteratively discussed and updated the data charting form as further themes emerged.

### Approach to results synthesis

Article demographics were summarized using descriptive statistics. To identify what post-licensure EPAs were reported and elucidate their nature, we extracted all EPAs in articles which were identified and referred to by the authors as “EPAs”. It was evident from the screening and data charting process that many articles referred to an EPA but reporting of any further specifications was absent or highly variable. We therefore engaged both thematic and narrative synthesis in an iterative approach to synthesize how EPAs were reported. We learned similar lessons to Thomas and colleagues [28] who reflect on the necessary subjectivist stance to define and organize the literature around clinical concepts with inherent multiple perspectives.

We took a stratified step-wise approach to synthesize how EPAs were reported according to the ten Cate 2005 attributes [5]. To feasibly manage the reported EPA volume, we limited our analysis to a selective sample of the first reported EPA in articles where more than one EPA was identified.

Based on charted data and reported themes, we mapped the first reported EPA in each article to the 8 specified concept attributes [5] which were published previous to all articles in our review.

EPA validity judgements were beyond the scope of this review, and heterogeneous EPA reporting rendered the otherwise relevant EPA quality assessment tools EQual [36] or QUEPA [37] tools unfeasible.

Undertaking a further thematic narrative synthesis, we explored whether translation of EPAs to practice could be feasible based on reported attributes and other details. This was based on the Tekian [22] perspective that reported EPAs may not be constructed according to the ten Cate attributes [5]. Since often only the EPA title was reported, we piloted and used Mulder’s [38] sentence-completing approach to understand whether EPAs could be interpretable from the title. E.g. Does it make sense when the EPA title is prefaced with "Can you perform…" and "Can you envisage the actual start and end of task?".

We also sought to synthesize a perspective on whether the first-reported EPA represented profession-specific skills or could have transdisciplinary relevance.

## Results

**Fig. 1:**
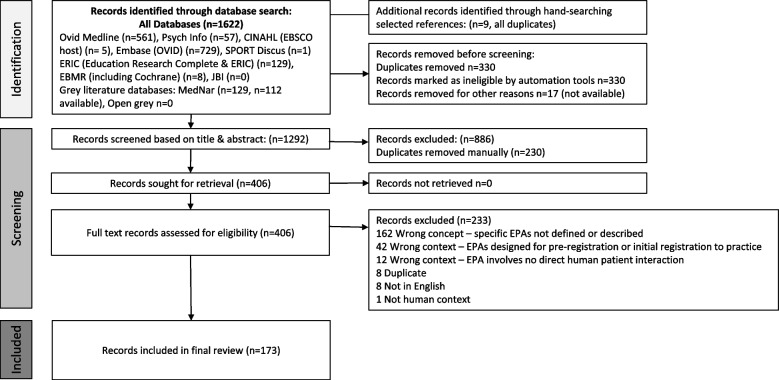
Review process and results—adapted PRISMA flow diagram. Abbreviations: Entrustable Professional Activity (EPA)

After duplicates were removed, our search identified 1622 articles and 173 articles were included in our final review. Figure 1 represents the PRISMA flow detailing our process of evidence identification and selection. Supplementary Table 2 contains a table and reference list of all included articles (*n* = 173) in this review.

Adopting the approach of Eva [34], herein we take a pragmatic and meaningful approach to present our critical narrative synthesis outcomes in the context of advancing the current perspective of post-licensure EPAs. Our findings are supported by, but not reproducing what is already known and represented by our large number of included articles (*n* = 173). All included article characteristics and demographics and reference list are reported with detailed tabulation in Supplementary Table 2 – Included article characteristics and reference list.

### Article characteristics

There is a growing trend to report post-licensure clinical practice expectations using the EPA concept. Publications were between 2007–2021 and proportionally in most recent years, including more than half in 2019–2021 (*n* = 82, 53%). Post-licensure EPAs are well reported in the North American context, and in the medical discipline. They are also reported by a range of countries and different disciplines world-wide. In total, 16 countries and 4 different multi-national collaborates had published EPAs, with the majority in North America (*n* = 126, 73%), specifically United States (*n* = 100, 58%) and Canada (*n* = 26, 15%). Article characteristics are summarised in Table 2. EPA reporting in medicine was overwhelmingly represented (*n* = 162, 94%), further differentiated into 40 medical sub-specialties. Non-medical professions represented were nursing (n = 6, 3%), pharmacy (*n* = 2, 1%), dentistry (*n* = 1, 1%), physiotherapy (*n* = 1, 1%) and respiratory therapy (*n* = 1, 1%). Article distribution according to clinical profession and medical specialty context is shown in Table 3.

**Table 2:** Article characteristics. This table reports the distribution of included articles (*n* = 173, 100%) according to article type, year of publication and demographic location. Supplementary Digital Appendix 3 contains a table and reference list of all included articles in this review

Article characteristic	No. articles	
	**n**	**(%)**
**Total**	**173**	**(100)**
**Article type**		
Case or cohort report	56	(32)
Commentary reviews	36	(21)
Delphi study	22	(13)
Conference abstract	22	(13)
Qualitative research	19	(11)
Professional guideline	6	(3)
Systematic review	2	(1)
Scoping review	1	(1)
Other:	9	(5)
**Year of publication**		
2021	30	(17)
2020	28	(22)
2019	24	(14)
2018	18	(10)
2017	15	(9)
2016	17	(10)
2015	8	(5)
2014	15	(9)
2013	5	(3)
2012	1	(1)
2011	1	(1)
2007	1	(1)
**Article location**		
United States	100	(58)
Canada	26	(15)
Netherlands	11	(6)
Australia	8	(5)
Germany	5	(3)
India	4	(2)
Singapore	2	(1)
Switzerland	2	(1)
UK	2	(1)
Ethiopia	1	(1)
Ireland	1	(1)
Iran	1	(1)
Italy	1	(1)
Japan	1	(1)
Nepal	1	(1)
Singapore	1	(1)
Multi-national -	6	(3)
Europe	3	
Australia and New Zealand	1	
Netherlands, USA, Aus	1	
United States and Canada	1

**Table 3:** Article distribution according to clinical profession and medical specialty context. This table reports the distribution of included articles (*n* = 173) according to clinical profession and reported EPA attributes of alignment with professional standards, primary clinical and/or education purpose, and evaluation. Articles in the medicine discipline are further represented according to sub-specialty, both as a proportion of the total number of included articles in the review (*n* = 173); and as a proportion of articles in medicine (*n* = 162, 94%). Abbreviations: Obstetrics and Gynaecology (Obs/gynae) Public Health & Preventative Medicine (PHMC); Physical Medicine and Rehabilitation (PM&R)

**EPA(s) attribute:**			**Aligned with professional standards**		**Clinical and/or education purpose**		**Evaluation reported**		
	**No. articles**		**No. articles**		**No. articles**		**No. articles**		
**Health discipline**	**n**	**(%)**	**n**	**(%)**	**n**	**(%)**	**n**	**(%)**	
Medicine—all specialties	162	(94)	122	75	159	92	28	17	
Nursing/Practitioner	6	(3)	4	67	6	100	0	0	
Pharmacy/Pharmacology	2	(1)	2	100	2	100	0	0	
Dentistry	1	(1)	1	100	1	100	0	0	
Physio/physical Therapy	1	(1)	0	0	1	100	1	1	
Respiratory Therapy	1	(1)	1	100	1	100	0	0	
**Medicine specialty**	**No. articles**		**% of Medicine**	**Medicine specialty**			**No. articles**		**% of Medicine**
	**n**	**(%)**					**n**	**(%)**	
Paediatrics	29	(17)	18	Community			1	(1)	1
Internal Medicine	18	(10)	11	Ear, Nose & Throat			1	(1)	1
Surgery	18	(10)	11	Geriatrics			1	(1)	1
Cross-specialty	15	(9)	9	Gastrointestinal			1	(1)	1
Emergency Medicine	9	(5)	6	Hospice and Palliative			1	(1)	1
Psychiatry	8	(5)	5	Pathology			1	(1)	1
Gastroenterology	7	(4)	4	Neurosurgery			1	(1)	1
Radiology	5	(3)	3	Otorhinolaryngology			1	(1)	1
Obs/gynae	4	(2)	2	Palliative			1	(1)	1
Other specialty	4	(2)	2	PM&R			1	(1)	1
Anaesthetics	4	(2)	2	Primary Care, Oral Health			1	(1)	1
Family Medicine	3	(2)	2	Neurology			1	(1)	1
General Practice	3	(2)	2	Radiation Oncology			1	(1)	1
Oncology	3	(2)	2	Resuscitation			1	(1)	1
Sports/Orthopaedics	3	(2)	2	Rheumatology			1	(1)	1
Intensive care	2	(1)	1	Stroke			1	(1)	1
Nephrology	2	(1)	1	Surgery Neuro-Oncology			1	(1)	1
Pulmonary & critical care	2	(1)	1	Telehealth			1	(1)	1
Adolescent	1	(1)	1	Urology			1	(1)	1
Cardiology	1	(1)	1	PHPM			1	(1)	1

### Reporting of EPAs according to the ten Cate attributes

Table 4 shows reporting of EPA attributes and details according to the 8 specified ten Cate attributes [5].

**Table 4:** Reporting of EPA attributes and details. This table contains EPA attributes and details reported in articles included in this review. This includes reporting of EPA attributes according to the ten Cate attributes [5]; and other reported details relevant to concept fidelity and translation to practice and education

**ten Cate EPA attributes** **[**5**]**		**Reported indicator**	**n (%)**
**1**	Are part of essential professional work in a given context	Reported in post-registration clinical health care context	173 (100)
**2**	Require adequate knowledge, skill and attitude, generally acquired through training Should usually be confined to qualified personnel	Reported in post-registration clinical health care context	173 (100)
**4**	Usually be confined to qualified personnel	Reported in post-registration clinical health care context Reported according to discipline-specific qualifications	173 (100) 138 (80)
**3**	Lead to recognized output of professional labor	Reported as aligned to professional competencies and/or standards	130 (75)
**8**	Reflect one or more of the competencies to be acquired	Reported sub-competencies or milestones	86 (50)
**7**	Observable and measurable in their process and their outcome, leading to a conclusion	Reported evaluation or assessment (including planned)	32 (18)
**5**	Independently executable	*Requires a validity judgement beyond the scope of this review*	
**6**	Executable within a timeframe	*Requires a validity judgement beyond the scope of this review*	
**Reporting of aggregate ten cate EPA attributes**			**n (%)**
*and*	Reported evaluation or assessment (including planned)		
*and*	Reported sub-competencies or milestones		
*and*	Reported as aligned to professional competencies and/or standards		10 (6)
**Other reported details**			**n (%)**
	Reported EPA design process		92 (53)
	- Reported as designed by a collaborative team of educators and clinicians		85 (49)
	EPA reported by title only		87 (50)
	EPA reported a combination of procedural and interpersonal skills		146 (84)
	Title wording represents an end-to-end task		84 (49)

All EPAs were reported to represent essential work confined to trained and qualified health professionals. Most reported content validation with specific alignment to discipline-recognized professional association standards (*n* = 130, 75%). Half of the articles further reported further specifications, for example discrete clinical skills required to perform the EPA or milestones to be acquired within EPAs (*n* = 86, 50%). The most infrequently reported attribute was any form of EPA construct evaluation or fit-for-purpose testing (*n* = 32, 18%).

### Narrative synthesis of EPA reporting

Our critical narrative synthesis of reported attributes arrived at four themes relating to how EPAs are reported:

(i) Many articles reported only EPA titles without further explanation.

Half the articles reported no further detail beyond EPA title(s) (*n* = 87, 50%). This precludes any validity judgement of EPA concept based on reporting of title alone, which was nevertheless beyond the scope of this review. We interpreted that half of reported EPAs had title wording where an end-to-end task could be envisaged (*n* = 84, 49%).

(ii) Many articles did not include information about the EPA design process.

Just over half the articles (*n* = 92, 53%) reported on the EPA design process, specifically involving educators (*n* = 1, 1%), clinicians (*n* = 5, 3%) or a collaborative team of educators and clinicians (*n* = 85, 49%). Half (*n* = 86, 50%) of the articles reported further EPA specifications, such as discrete clinical skills required to perform the EPA. Our review was unable to differentiate between EPAs which were briefly reported versus insufficiently constructed according to the ten Cate attributes [5].

(iii) Few EPAs and frameworks were reported according to all or most ten Cate[5] recommended EPA attributes

While all EPAs were reported according to several essential attributes through the inclusion criteria of this review, the proportion decreased when reported attributes were aggregated. Very few articles reported all the following details: alignment to professional competencies and standards, sub-competencies or milestones and evaluation or assessment (*n* = 10, 6%).

(iv) Limited attribute reporting precludes distinction between profession-specific EPAs and EPAs which could have transdisciplinary relevance

Most articles reported EPAs requiring discipline-specific qualifications (*n* = 138, 80%). The majority of EPAs also represented work-units requiring performance of a combination of procedural and interpersonal skills (*n* = 146, 84%). Our review did not discover any articles reporting EPAs as transdisciplinary. 

## Discussion

This scoping review addressed our initial inquiry purpose to explore how post-licensure EPAs are reported in peer reviewed literature, in different clinical professions. We identified a large, heterogeneous and evolving body of literature which extends upon previous scoping reviews describing the increasing utilization of EPAs in pre-licensure (entry-to-practice) healthcare or medicine only [6, 9]. Further to themes identified in the results, here we discuss the current landscape of post-licensure EPAs and how they are reported. We embrace Eva’s [34] intention to advance the perspective by advocating application to practice of well-established knowledge, highlighting the disparity in EPA volume in medicine compared to other clinical disciplines and synthesizing recommendations to overcome the identified problem of heterogeneous reporting. Importantly, we based our enquiry upon existing EPA guidelines and specifications [5, 21, 39]. Indeed our experience in conducting the review and primary finding was the insufficiency and heterogeneity in how post-licensure EPAs are reported in relation to these.

### EPA concept and reporting specifications

Our scoping review offers a pragmatic perspective including problems evident in the literature and reporting of current post-licensure EPAs. Our review encountered and navigated challenges, inconsistencies and non-specific EPA terminology, which is consistent with other contemporary perspectives around EPA concept conflation and viability [19, 40, 41]. Our review does not redress the nuance of language or conflicting terminology relating to existing EPAs, rather we emphasise that this was also evident in our review. This gave rise to methodological challenges and highlighted the need for nuanced interpretation of EPAs according to the intended purpose and context. Accordingly, we emphasize the limiting impacts of such necessary subjective EPA interpretation, which also has implications for transparent application for other EPA consumers in practice and education. Subsequent to our review, Hennus and colleagues [40] also acknowledge mixed logic and rationale for EPA development and design, including significant existence of “non-EPAs” which are not constructed in a way which makes them suitable for entrustment decisions.

It was beyond the scope of this review to make validity judgments of EPAs based on only reported attributes and details, moreover when often the title alone was reported. It is not feasible to judge EPA construct and validity from the reporting if EPAs are reported without the full specifications. Nevertheless, it is pertinent to differentiate whether any critical insufficiency lies within the EPA construct or in reporting, also recognizing that either will impact fidelity and translation to practice.

Our review demonstrates that many post-licensure EPAs are not reported according to contemporary construct recommendations, and demonstrates the problematic concerns and inconsistencies highlighted in existing literature.

### How post-licensure EPAs are reported

This is the first review focused on contextualizing the volume and reporting of post-licensure EPAs including both medical and non-medicine clinical professions. In a rapidly evolving field, our review captured a large volume of EPAs reported in medicine, proportionally well established in North America but also utilized by a range of nations worldwide. Specifically, these work-units were to be entrusted during and/or at end-of-training medical specialization fellowships, aligned with and beyond the CanMEDS transition to discipline stage towards advanced expertise [25]. Other healthcare disciplines with published post-licensure EPAs were nursing, pharmacy, dentistry, physiotherapy and respiratory therapy.

Discovering the volume of post-licensure medical EPAs alongside the relative few available in other healthcare professions extends on the perspective offered by previous reviews by O’Dowd et al. [17] and Lui et al. [18] from several perspectives. Our broader search and inclusion lens, substantiated with our finding that often only the EPA title was reported (without further specifications or attributes), demonstrates that the breadth and depth of existing EPAs is more extensive than revealed in previous reviews. We found that most EPAs are not reported with sufficient further specifications to support fidelity or translation to practice based on reporting. We speculate that insufficiently reported EPAs may not have met the inclusion criteria for exploration or analysis in previous reviews.

Another important point of difference to these previous reviews was our aim to identify any potentially transdisciplinary EPAs. Although EPAs are conventionally discipline-specific rather than interprofessional [19], the concept and value of transdisciplinary EPAs has been recognized subsequent to this review [20]. The majority of articles in our review reported EPAs requiring a combination of procedural and interpersonal skills, some of which could plausibly be relevant to clinicians in different professions or across disciplines. However, without reporting of full EPA specifications, any judgement of transdisciplinary relevance based on reporting risks being misguided.

In the context of other evidence our findings support the widely reported perspective of rapidly expanding EPA reporting for the primary purpose of aligning clinical skill education and practice [6, 9, 39]. Bramley and colleagues’ scoping review of pre-licensure healthcare EPAs similarly described EPAs from the United States, Canada, Europe, Australia and Central America; in different disciplines of medicine, pharmacy, dietetics and physician assistants [9]. Shorey and colleagues’ scoping review reported their eighty included articles describing EPAs in healthcare education were also weighted unequally across geographic regions and lacked high quality evidence [6].

Articles in our review often reported EPAs by title only. Established recommendations for EPA construct include unambiguous titles [5, 16] and common taxonomy [42] to avoid linguistic confusion [43]. That said, contemporary guidelines acknowledge formulating a concise EPA-defining title can present linguistic challenges, compromise EPA breadth and is not essential if other specifications capture the EPA framework clearly [39, 44]. Notwithstanding whether EPAs were designed according to recommended EPA construct principles [5, 39], these further specifications were inconsistently reported.

Placing the importance of all EPA specifications and attributes in perspective, EPAs are intended to represent task-integrated performance of essential clinical skills defined in health professionals' competency standards matrices [5]. This purposeful alignment was reported in half the articles in our review. Some also reported a rigorous, collaborative design process involving both clinicians and educators while often the rigour of design process was unclear. We acknowledge the reporting context can curtail the level of detail presented (e.g. conference abstracts, article focus and word limits).

Despite incomplete reporting of EPA attributes, most EPAs were reported as aligned to relevant benchmarked clinical tasks endorsed by discipline-specific professional associations and/or content validated with alignment to professional competency matrices [21]. This indicates most EPAs are reported as representing the task-integration of validated skills required of post-licensure clinicians.

### Implications for practice, policy and recommendations

Our review determined that most EPAs were reported as designed according to professional competency matrices, but without reporting any fit-for-purpose evaluation. Alongside ambiguous task definitions, this risks fidelity in the formation of ad-hoc expectations, and inequitable learning experience and assessment of essential skills. Further, the extent of EPA implementation and evaluation is integral to determining fit-for-purpose [44]. Since so few articles reported EPA evaluation of any kind, how post-licensure EPAs meet their intended purpose of aligning clinical skill education and practice could not be determined from the reporting.

Resolving inconsistency of EPA reporting would facilitate transparent real-world implementation for consumers in clinical education and clinical practice. Advancing popularity and EPA concept dilution led ten Cate & Taylor [39] to further clarify EPA features and structure; and ten Cate & Chen [21] to recommend specific approaches to content-validate EPAs including matrix-map-to-competency frameworks. The nature of literature in our review validates contemporary concerns about diverse EPA descriptions, demonstrating the problems encountered in deciphering EPAs with content dilution and/or when established recommendations are sporadically reported. Our experience substantiates the worth of advancing the practice approach to rigorous EPA reporting towards concept fidelity and validating design integrity.

Advocating, synthesizing and reporting future EPAs according to the current existing recommendations underpins our further three recommendations based on our experience of interpreting how post-licensure EPAs are reported in peer reviewed literature. We discovered a vast number of EPAs representing post-licensure clinical practice in different healthcare disciplines and acknowledge the variety of EPA reporting contexts in our review. To promote EPA fidelity, quality appraisal and reduce subjectivity in consumer interpretation, we assert the proportional value of the following recommendations towards reporting EPA features and attributes:

1. Diligently reporting EPAs with their attributes and features, or directly citing towards the full specifications.
2. Including reference to EPA design and content-validity information.
3. Distinguishing the EPA as profession-specific or transdisciplinary relevant

Previously published recommendations for EPA construct and reporting in the context of our further reporting recommendations are represented in Supplementary file 1.

### Limitations of included evidence and the review process

Our review included EPAs referred to as EPAs by article authors in peer-reviewed literature. Some EPAs were reported in several different articles, so our *n* value represents reporting volume, with some EPAs in duplicate. We grounded a feasible-sized synthesis using the first reported EPA title in each article, so further examples of high-integrity EPAs may have been omitted. It was beyond our resource to consider the large volume of heterogeneous evidence using a consistent Quality Appraisal Tool, which would have been valuable for commenting on the overall rigour of available evidence. Our review included all post-licensure EPAs, without discerning the organization of EPAs along the staged continuum of post-licensure professional training. This review can inform identification of focus points and approach for future work.

### Reflexivity statement

Instead of optional additional stakeholder consultation, we drew upon the varied expertise within our research team. We prospectively acknowledged the subjectivity of our scoping review methodology, and further extend this to our critical narrative perspective and interpretation of EPAs. We acknowledge any influence of the wholly physiotherapy-discipline authorship, including any prior assumptions, beliefs and experience as practicing physiotherapists, active researchers and interprofessional education scholars. Reciprocally, we attest our combined and different extensive experiences in these fields qualify us to perform this review, leveraging our combined and different scholarly perspectives to formulate a valid perspective. We attest to the rigorous design and transparent reporting of our work. We developed our conclusions and recommendations with these limitations in mind, advocating the review size enabled us to demonstrate the large heterogenous body of literature and a robust subjective overview of the current evidence base.

## Conclusion

EPAs are an established approach to capturing post-licensure clinical skill requirements in different healthcare professions and extensively in medical specialist training. EPAs are typically designed to align clinical skill education and practice according to professional competency standards matrices. In answer to our research question, a large volume of post-licensure EPAs is reported in medicine and very few in other clinical professions. EPA specifications beyond the title were absent or variously reported in the literature, risking ambiguous interpretation of the education and practice expectations. We recommend that future EPAs are reported with reference to established and evolving construct recommendations, which is integral to concept fidelity and translation to practice and education.

## Supplementary Information

**Additional  file 1.** 

**Additional  file 2.** 

**Additional  file 3.** 

**Additional  file 4.** 

## Data Availability

All data generated or analysed during this study are included in this published article and its supplementary information files.

## Abbreviations

EPA: Entrustable Professional Activity

## References

[1] Michels ME, Evans DE, Blok GA (2012). What is a clinical skill? Searching for order in chaos through a modified Delphi process. Med Teach.

[2] Ten Cate O, Tobin S, Stokes ML (2017). Bringing competencies closer to day-to-day clinical work through entrustable professional activities. Med J Aust.

[3] AHPRA. Australian Health Professionals Registration Agency (AHPRA) standards. https://www.ahpra.gov.au/Registration/Registration-Standards.aspx. Accessed Nov 2021.

[4] AMC. Australian Medical Council (AMC) Assessment & accreditation of specialist medical programs. https://www.amc.org.au/accreditation-and-recognition/assessment-accreditation-specialist-medical-programs-assessment-accreditation-specialist-medical-programs/. Accessed Nov 2021.

[5] ten Cate O (2005). Entrustability of professional activities and competency-based training. Med Educ.

[6] Shorey S, Lau TC, Lau ST, Ang E (2019). Entrustable professional activities in health care education: a scoping review. Med Educ.

[7] ten Cate O, Scheele F (2007). Competency-based postgraduate training: can we bridge the gap between theory and clinical practice?. Acad Med.

[8] White K, Qualtieri J, Courville EL, Beck RC, Alobeid B, Czuchlewski DR (2021). Entrustable Professional Activities in Hematopathology Pathology Fellowship Training: Consensus Design and Proposal. Acad Pathol.

[9] Bramley AL, McKenna L (2021). Entrustable professional activities in entry-level health professional education: A scoping review. Med Educ.

[10] Neumann PB, Radi N, Gerdis TL, Tonkin C, Wright C, Chalmers KJ (2022). Development of a multinational, multidisciplinary competency framework for physiotherapy training in pessary management: an E-Delphi study. Int Urogynecol J.

[11] Ryan MS, Richards A, Perera R, Park YS, Stringer JK, Waterhouse E, et al. Generalizability of the Ottawa Surgical Competency Operating Room Evaluation (O-SCORE) scale to assess medical student performance on core EPAs in the workplace: findings from one institution. Acad Med. 2021. 10.1097/ACM.0000000000003921 33464735

[12] Soukoulis V, Martindale J, Bray MJ, Bradley E, Gusic ME (2021). The use of EPA assessments in decision-making: Do supervision ratings correlate with other measures of clinical performance?. Med Teach.

[13] Zainuldin R, Tan HY (2021). Development of entrustable professional activities for a physiotherapy undergraduate programme in Singapore. Physiotherapy.

[14] AQF. Australian Qualifications Framework (AQF) Levels. https://www.aqf.edu.au/aqf-levels. Accessed Nov 2021.

[15] Chesbro SB, Jensen GM, Boissonnault WG (2018). Entrustable Professional Activities as a Framework for Continued Professional Competence: Is Now the Time?. Phys Ther.

[16] Ten Cate O, Carraccio C, Damodaran A, Gofton W, Hamstra SJ, Hart DE (2021). Entrustment Decision Making: Extending Miller's Pyramid. Acad Med.

[17] O'Dowd E, Lydon S, O'Connor P, Madden C, Byrne D (2019). A systematic review of 7 years of research on entrustable professional activities in graduate medical education, 2011–2018. Med Educ.

[18] Liu L, Jiang Z, Qi X, Xie A, Wu H, Cheng H (2021). An update on current EPAs in graduate medical education: A scoping review. Med Educ Online.

[19] Ten Cate O, Pool IA (2020). The viability of interprofessional entrustable professional activities. Adv Health Sci Educ Theory Pract.

[20] Pool I, Hofstra S, van der Horst M, Ten Cate O. Transdisciplinary entrustable professional activities. Med Teach. 2023:1–6. 10.1080/0142159X.2023.2170778 36708704

[21] Ten Cate O, Chen HC, Hoff RG, Peters H, Bok H, van der Schaaf M. Curriculum development for the workplace using Entrustable Professional Activities (EPAs): AMEE Guide No. 99. Med Teach. 2015;37(11):983–1002. 10.3109/0142159X.2015.1060308 26172347

[22] Tekian A (2017). Are all EPAs really EPAs?. Med Teach.

[23] Group ML. Medical Licensure Group. https://medicallicensuregroup.com/when-to-get-medical-license/. Accessed Apr 2023.

[24] Canada RCoPaSo. CanMEDS: Better standards, better physicians, better care. https://www.royalcollege.ca/rcsite/canmeds/canmeds-framework-e#:~:text=CanMEDS%20is%20a%20framework%20that,The%20CanMEDS%20Roles. Accessed Apr 2023.

[25] CanMEDS. CanMEDS Guide: Royal College Canada. https://canmeds.royalcollege.ca/guide. Accessed Apr 2023.

[26] Warm EJ, Kinnear B, Lance S, Schauer DP, Brenner J (2022). What Behaviors Define a Good Physician? Assessing and Communicating About Noncognitive Skills. Acad Med.

[27] Thomas A, Lubarsky S, Durning SJ, Young ME (2017). Knowledge Syntheses in Medical Education: Demystifying Scoping Reviews. Acad Med.

[28] Thomas A, Lubarsky S, Varpio L, Durning SJ, Young ME (2020). Scoping reviews in health professions education: challenges, considerations and lessons learned about epistemology and methodology. Adv Health Sci Educ Theory Pract.

[29] Tricco AC, Lillie E, Zarin W, O'Brien KK, Colquhoun H, Levac D (2018). PRISMA Extension for Scoping Reviews (PRISMA-ScR): Checklist and Explanation. Ann Intern Med.

[30] Arksey H, O'Malley L (2005). Scoping studies: towards a methodological framework. Int J Soc Res Methodol.

[31] Aromataris EM, Z. JBI Manual for Evidence Synthesis: JBI. https://synthesismanual.jbi.global. 10.46658/JBIMES-20-01. Accessed Nov 2021.

[32] Levac D, Colquhoun H, O'Brien KK (2010). Scoping studies: advancing the methodology. Implement Sci.

[33] Moore S, Egerton T, La Scala N, Merolli M, Lees J, Parry S. Entrustable professional activities (EPAs) in post-registration healthcare practice and education: a scoping review protocol. https://osf.io/q5ty8. Accessed Apr 2023.

[34] Eva KW (2008). On the limits of systematicity. Med Educ.

[35] Innovation VH. Covidence systematic review software. Melbourne, Australia2022.

[36] Taylor D, Park YS, Egan R, Chan M-KK, Jolanta;, Touchie CS, Linda; Tekian, Ara. EQual, a Novel Rubric to Evaluate Entrustable Professional Activities for Quality and Structure. Academic Medicine. 2017;92(11S) Association of American Medical Colleges Learn Serve(Lead):Proceedings of the 56th Annual Research in Medical Education Sessions:S110-S7. 10.1097/ACM.0000000000001908 29065031

[37] Post JA, Wittich CM, Thomas KG, Dupras DM, Halvorsen AJ, Mandrekar JN (2016). Rating the Quality of Entrustable Professional Activities: Content Validation and Associations with the Clinical Context. J Gen Intern Med.

[38] Mulder H, Ten Cate O, Daalder R, Berkvens J (2010). Building a competency-based workplace curriculum around entrustable professional activities: The case of physician assistant training. Med Teach.

[39] Ten Cate O, Taylor DR. The recommended description of an entrustable professional activity: AMEE Guide No. 140. Med Teach. 2021;43(10):1106–14. 10.1080/0142159X.2020.1838465 33167763

[40] Hennus MP, van Dam M, Gauthier S, Taylor DR, Ten Cate O (2022). The logic behind entrustable professional activity frameworks: A scoping review of the literature. Med Educ.

[41] Ten Cate O, Schumacher DJ (2022). Entrustable professional activities versus competencies and skills: Exploring why different concepts are often conflated. Adv Health Sci Educ Theory Pract.

[42] Englander R, Cameron T, Ballard AJ, Dodge J, Bull J, Aschenbrener CA (2013). Toward a common taxonomy of competency domains for the health professions and competencies for physicians. Acad Med.

[43] Lohenry KC, Brenneman A, Goldgar C, Hills KJ, VanderMeulen SP, Lane S (2017). Entrustable Professional Activities: A New Direction for PA Education?. J Physician Assist Educ.

[44] Hennus MP, Jarrett JB, Taylor DR, Ten Cate O. Twelve tips to develop entrustable professional activities. Med Teach. 2023:1–7. 10.1080/0142159X.2023.2197137 37027517

