# Supplementary material for: Inconsistently reporting post-licensure EPA specifications in different clinical professions hampers fidelity and practice translation: a scoping review
Source: BMC Med Educ. 2023 May 24;23:372. doi: 10.1186/s12909-023-04364-4 (PMC10207741; doi:10.1186/s12909-023-04364-4)
Supplement: Supplementary file 1 — Additional file 1. [file 12909_2023_4364_MOESM1_ESM.pptx]

## Slide 1
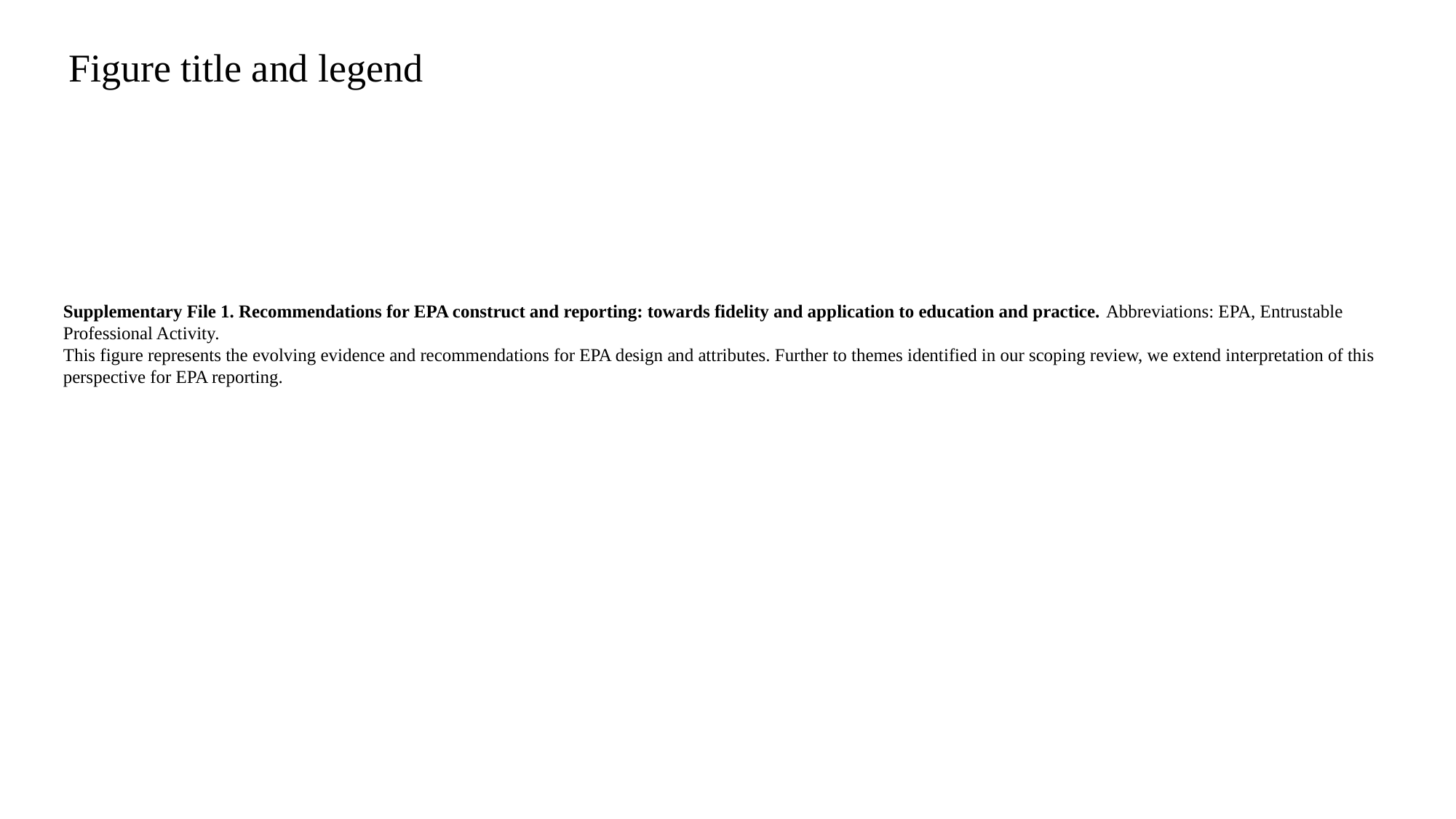

Figure title and legend
Supplementary File 1. Recommendations for EPA construct and reporting: towards fidelity and application to education and practice. Abbreviations: EPA, Entrustable Professional Activity.
This figure represents the evolving evidence and recommendations for EPA design and attributes. Further to themes identified in our scoping review, we extend interpretation of this perspective for EPA reporting.

## Slide 2
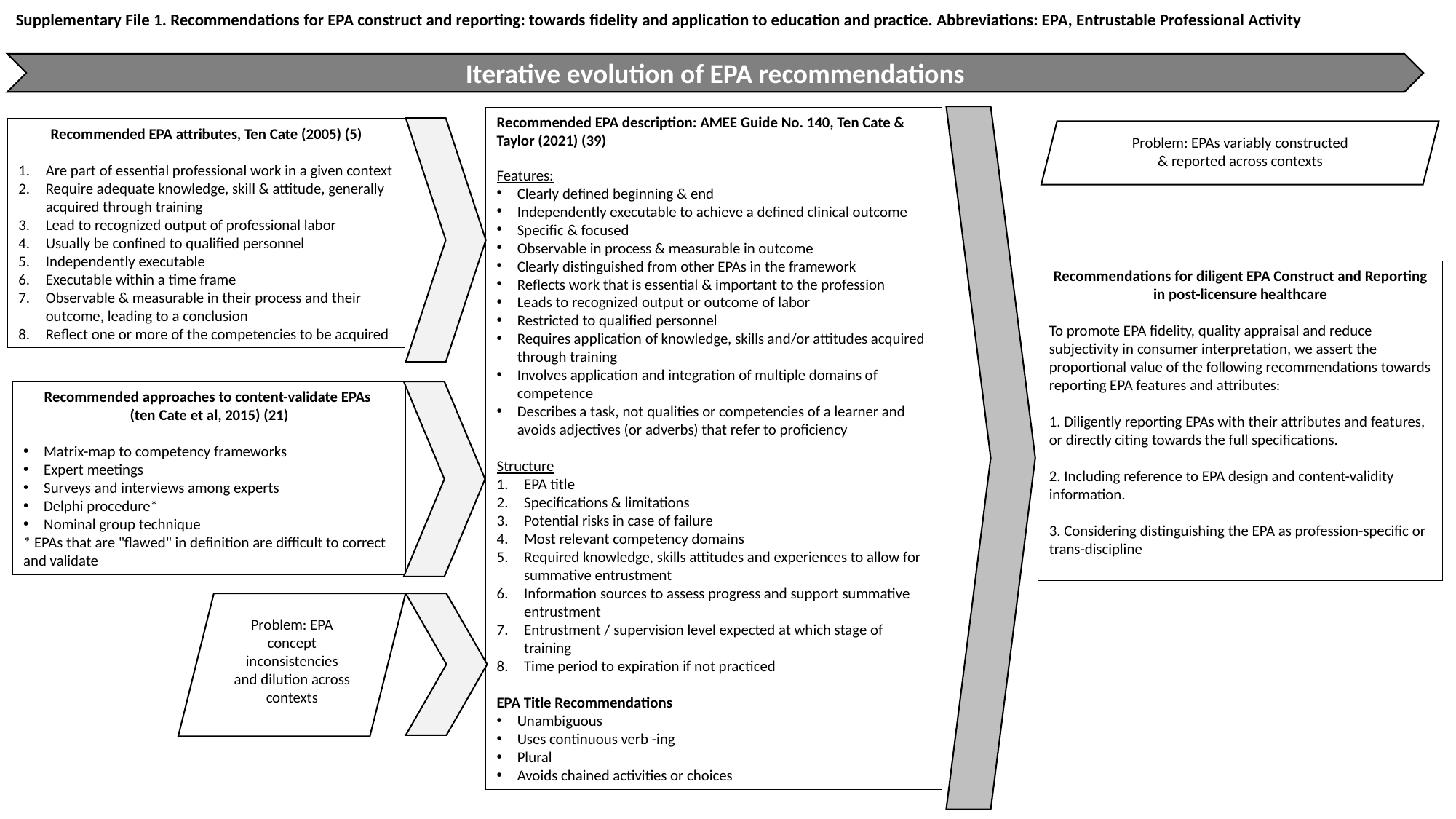

Supplementary File 1. Recommendations for EPA construct and reporting: towards fidelity and application to education and practice. Abbreviations: EPA, Entrustable Professional Activity
Iterative evolution of EPA recommendations
Recommended EPA description: AMEE Guide No. 140, Ten Cate & Taylor (2021) (39)
Features:
Clearly defined beginning & end
Independently executable to achieve a defined clinical outcome
Specific & focused
Observable in process & measurable in outcome
Clearly distinguished from other EPAs in the framework
Reflects work that is essential & important to the profession
Leads to recognized output or outcome of labor
Restricted to qualified personnel
Requires application of knowledge, skills and/or attitudes acquired through training
Involves application and integration of multiple domains of competence
Describes a task, not qualities or competencies of a learner and avoids adjectives (or adverbs) that refer to proficiency
Structure
EPA title
Specifications & limitations
Potential risks in case of failure
Most relevant competency domains
Required knowledge, skills attitudes and experiences to allow for summative entrustment
Information sources to assess progress and support summative entrustment
Entrustment / supervision level expected at which stage of training
Time period to expiration if not practiced
EPA Title Recommendations
Unambiguous
Uses continuous verb -ing
Plural
Avoids chained activities or choices
Recommended EPA attributes, Ten Cate (2005) (5)
Are part of essential professional work in a given context
Require adequate knowledge, skill & attitude, generally acquired through training
Lead to recognized output of professional labor
Usually be confined to qualified personnel
Independently executable
Executable within a time frame
Observable & measurable in their process and their outcome, leading to a conclusion
Reflect one or more of the competencies to be acquired
Problem: EPAs variably constructed & reported across contexts
Recommendations for diligent EPA Construct and Reporting in post-licensure healthcare
To promote EPA fidelity, quality appraisal and reduce subjectivity in consumer interpretation, we assert the proportional value of the following recommendations towards reporting EPA features and attributes:
1. Diligently reporting EPAs with their attributes and features, or directly citing towards the full specifications.
2. Including reference to EPA design and content-validity information.
3. Considering distinguishing the EPA as profession-specific or trans-discipline
Recommended approaches to content-validate EPAs
(ten Cate et al, 2015) (21)
Matrix-map to competency frameworks
Expert meetings
Surveys and interviews among experts
Delphi procedure*
Nominal group technique
* EPAs that are "flawed" in definition are difficult to correct and validate
Problem: EPA concept inconsistencies and dilution across contexts
