# Supplementary material for: Inconsistently reporting post-licensure EPA specifications in different clinical professions hampers fidelity and practice translation: a scoping review
Source: BMC Med Educ. 2023 May 24;23:372. doi: 10.1186/s12909-023-04364-4 (PMC10207741; doi:10.1186/s12909-023-04364-4)
Supplement: Supplementary file 2 — Additional file 2. [file 12909_2023_4364_MOESM2_ESM.docx]

**Supplementary file - Data charting template**

# **General information**

**Authors**

List surname of all authors separated by a comma, using "&" prior to the final author.

**Year of publication**

# **Characteristics of included studies**

**Country of source context / study conducted**

- Australia
- Canada
- Ethiopia
- Germany
- India
- Ireland
- Guatemala
- Nepal
- Netherlands
- Singapore
- UK
- United States
- Other

**Study design**

- Delphi study
- Systematic review
- Scoping review
- Meeting abstract
- Conference abstract
- Professional guideline document
- Cohort study
- Commentary, review, letter or opinion
- Qualitative research
- Case report
- Other

# **Context**

**Post-registration clinical discipline**

- Audiology
- Dentistry
- Medicine - Anaesthetics/anaesthesiology
- Medicine - Emergency Medicine
- Medicine - Intensive care
- Medicine - Internal
- Medicine - GP
- Medicine - Obs/gynae
- Medicine - Paediatrics
- Medicine - Pathology
- Medicine - Physical Medicine & Rehabilitation
- Medicine - Psychiatry
- Medicine - Radiology
- Medicine - Sports/MSK/Orthopaedics
- Medicine - Surgery
- Medicine - Cross-specialty / within scope of practice/training
- Medicine - Other post-qualification specialty
- Midwifery
- Nursing / Nurse Practitioner
- Occupational therapy
- Optometry
- Osteopathy
- Pharmacy/Pharmacology
- Physiotherapy/Physical Therapy
- Podiatry
- Psychology
- Respiratory Therapist
- Social work
- Speech pathology
- Other

**Number of EPAs reported**

- 0
- 1
- 2
- 3
- 4
- 5
- 6
- 7
- 8
- 9
- 10
- 11
- 12
- 13
- 14
- 15
- 16
- 17
- 18
- 19
- 20
- 21
- 22
- 23
- 24
- 25
- 26
- 27
- 28
- 29
- 30
- 31
- 32
- 33
- 34
- 35
- 36
- 37
- 38
- 39
- 40
- 41
- 42
- 43
- 44
- 45
- 46
- 47
- 48
- 49
- 50
- more than 50
- Other

**Intended purpose**

What is the primary intended purpose of the research activity or discussion within the source?

- Design new EPA(s)
- Define / describe existing EPA(s)
- Evaluation of EPA(s)
- EPA application - clinical practice context
- EPA application - education context
- EPA application - both clinical and education context
- Other

**Aims of study**

**Main outcomes**

What were the primary outcomes of the study? (e.g. n EPAs were designed; X EPA was evaluated and main findings were...; y EPA was discussed.)

# **Concept: Reported EPA(s)**

**EPA title(s) or definition**

1. Capture the overarching title of each EPA identified in the source

2. If specified in the source, note in as much detail as is available the cohort of clinicians the EPA applies to. (eg. residency; PGY1, PGY2, PGY3, PGY4, PGY5; foundation residency training, progressive residency training with different milestones according to year; fellowship.)

|  | **EPA title (or group if more than 50)** | **Cohort of clinicians EPA is applicable to** |
| --- | --- | --- |
| **EPA 1** |  |  |
| **EPA 2** |  |  |
| **EPA 3** |  |  |
| **EPA 4** |  |  |
| **EPA 5** |  |  |
| **EPA 6** |  |  |
| **EPA 7** |  |  |
| **EPA 8** |  |  |
| **EPA 9** |  |  |
| **EPA 10** |  |  |
| **EPA 11** |  |  |
| **EPA 12** |  |  |
| **EPA 13** |  |  |
| **EPA 14** |  |  |
| **EPA 15** |  |  |
| **EPA 16** |  |  |
| **EPA 17** |  |  |
| **EPA 18** |  |  |
| **EPA 19** |  |  |
| **EPA 20** |  |  |
| **EPA 21** |  |  |
| **EPA 22** |  |  |
| **EPA 23** |  |  |
| **EPA 24** |  |  |
| **EPA 25** |  |  |
| **EPA 26** |  |  |
| **EPA 27** |  |  |
| **EPA 28** |  |  |
| **EPA 29** |  |  |
| **EPA 30** |  |  |
| **EPA 31** |  |  |
| **EPA 32** |  |  |
| **EPA 33** |  |  |
| **EPA 34** |  |  |
| **EPA 35** |  |  |
| **EPA 36** |  |  |
| **EPA 37** |  |  |
| **EPA 38** |  |  |
| **EPA 39** |  |  |
| **EPA 40** |  |  |
| **EPA 41** |  |  |
| **EPA 42** |  |  |
| **EPA 43** |  |  |
| **EPA 44** |  |  |
| **EPA 45** |  |  |
| **EPA 46** |  |  |
| **EPA 47** |  |  |
| **EPA 48** |  |  |
| **EPA 49** |  |  |
| **EPA 50** |  |  |

**OPTIONAL: If there are more than 50 EPAs identified, chart how many there are and how they are organised (eg subheadings, topics.)**

Add any further descriptive information that describes how the EPAs are reported. (eg. an extensive list of 200 EPAs is in Appendix X)

**For the EPAs identified, are any embedded clinical skills, competencies or milestones identified in further detail?**

- Constituent clinical skills
- Milestones
- Competencies
- No

**OPTIONAL: If reported, chart any clinical skills, competencies or milestones identified within each EPA**

Record the EPA title. Identify each Tier 1 constituent clinical skills within the EPA. (eg key skills, main headings.) For each Tier 1 clinical skill, list any 2nd tier constituent clinical skills described. (eg further sub-descriptors or behavioural indicators, sub-heading categories.)

|  | **EPA 1** | **EPA 2** | **EPA 3** | **EPA 4** | **EPA 5** | **EPA 6** | **EPA 7** | **EPA 8** | **EPA 9** | **EPA 10** |
| --- | --- | --- | --- | --- | --- | --- | --- | --- | --- | --- |
| **EPA title** |  |  |  |  |  |  |  |  |  |  |
| **Constituent clinical skill/competency/milestone 1** |  |  |  |  |  |  |  |  |  |  |
| **Constituent clinical skill/competency/milestone 2** |  |  |  |  |  |  |  |  |  |  |
| **Constituent clinical skill/competency/milestonel 3** |  |  |  |  |  |  |  |  |  |  |
| **Constituent clinical skill/competency/milestone 4** |  |  |  |  |  |  |  |  |  |  |
| **Constituent clinical skill/competency/milestone 5** |  |  |  |  |  |  |  |  |  |  |
| **Constituent clinical skill/competency/milestone 6** |  |  |  |  |  |  |  |  |  |  |
| **Constituent clinical skill/competency/milestone 7** |  |  |  |  |  |  |  |  |  |  |
| **Constituent clinical skill/competency/milestone 8** |  |  |  |  |  |  |  |  |  |  |
| **Constituent clinical skill/competency/milestone 9** |  |  |  |  |  |  |  |  |  |  |
| **Constituent clinical skill/competency/milestone 10** |  |  |  |  |  |  |  |  |  |  |

**Besides the overarching title, how many tiers of constituent clinical skills are reported to define the EPA?**

- None
- 1
- 2
- 3
- 4
- 5
- more than 5

**The MAIN focus of the EPA(s) (or set of EPAs reported) is (choose one):**

- Manual/procedural clinical skill(s) eg medical procedure, hand washing, manual technique
- Interpersonal clinical skill(s) eg communication, management
- Integration of manual/procedural and interpersonal clinical skills
- One or the other of manual/procedural and interpersonal skills, if there is a list of different EPAs identified
- Unclear / insufficient detail

# **EPA(s) design, development, implementation and evaluation (if applicable to source)**

**EPA(s) designed by:**

- Educator(s)
- Clinician(s)
- Academic(s)/Researcher(s)
- Collaborate team of two or more of the above
- Unclear / insufficient detail
- Other

**EPA(s) primary intended purpose**

- To capture / describe clinical practice expectation(s)
- To inform education / curriculum design
- To inform clinical skill assessment
- To inform BOTH education / curriculum design and clinical skill assessment
- To be implemented in real-world clinical practice
- Unclear / insufficient detail
- Other

**Comment on primary development/implementation purpose (optional)**

**Has the EPA(s) been implemented?**

- Yes
- No
- Implementation not reported in this source
- Planned
- In progress
- Unclear / insufficient detail

**Comment on implementation (optional)**

**Has the EPA(s) been assessed or evaluated? (ie a fit-for purpose or implementation appraisal?)**

- Yes
- Assessment or evaluation not reported in this source
- Planned
- In progress
- Unclear / insufficient detail

**Comment on assessment / evaluation (optional). If yes, provide process and outcome**

## **Alignment to endorsed practice requirements**

**Is / was the EPA(s) designed specifically to align with peer endorsed practice requirements? (eg recognised professional standards)**

- Yes
- No
- Not reported as such
- Unclear / insufficient detail

**Is / was the EPA(s) formally endorsed or validated by professional organisation(s)?**

- Yes
- No
- Planned
- In progress
- Unclear / insufficient detail

**Comment on alignment to endorsed practice requirements (optional). If yes - which organisation?**

**This source presents EPA(s) within the context of a research design and/or with sufficient detail suitable for Quality Assessment using an appropriate tool.**

- Yes
- No
