# Supplementary material for: Inconsistently reporting post-licensure EPA specifications in different clinical professions hampers fidelity and practice translation: a scoping review
Source: BMC Med Educ. 2023 May 24;23:372. doi: 10.1186/s12909-023-04364-4 (PMC10207741; doi:10.1186/s12909-023-04364-4)
Supplement: Supplementary file 3 — Additional file 3. [file 12909_2023_4364_MOESM3_ESM.docx]

**Supplementary Table 1**

**MEDLINE search strategy**

| 1 | Entrustable professional activit*.mp |
| --- | --- |
| 2 | (Dental practice or dentistry).mp |
| 3 | (Medical practice or GP or general practi* or doctor or physician).mp |
| 4 | (Medical radiation practice or radiography or radiation therapy).mp |
| 5 | (Nurse* or nursing).mp |
| 6 | (Midwife* or midwives).mp |
| 7 | Occupational therap*.mp |
| 8 | (Optometry or optometrist*).mp |
| 9 | Osteopath*.mp. |
| 10 | Pharmacology.mp |
| 11 | Physiotherap*.mp |
| 12 | Podiatry.mp |
| 13 | Psycholog*.mp |
| 14 | allied health professional.mp |
| 15 | clinical practice.mp |
| 16 | audiolog*.mp |
| 17 | social work.mp |
| 18 | speech patholog*.mp |
| 19 | (clinic* or health*) |
| 20 | 2 or 3 or 4 or 5 or 6 or 7 or 8 or 9 or 10 or 11 or 12 or 13 or 14 or 15 or 16 or 17 or 18 or 19 |
| 21 | 1 and 20 |
