# Supplementary material for: Inconsistently reporting post-licensure EPA specifications in different clinical professions hampers fidelity and practice translation: a scoping review
Source: BMC Med Educ. 2023 May 24;23:372. doi: 10.1186/s12909-023-04364-4 (PMC10207741; doi:10.1186/s12909-023-04364-4)
Supplement: Supplementary file 4 — Additional file 4. [file 12909_2023_4364_MOESM4_ESM.docx]

**Supplementary Table 2 – Included articles and reference list**

**Included articles (n=173)**

|  | **Country context of article** | **Article design** | **Post-registration clinical discipline** | **No. EPAs reported** | **Aligned with prof-essional competency standards** | **Unambiguous EPA Title** | **Competency domains or milestones described** | **EPA potentially relevant to all clinicians** | **Evaluation reported** | **EPA(s) designed by:** | **Integrated practical and interpersonal skills** | **Primary purpose for clinical and/or education** |
| --- | --- | --- | --- | --- | --- | --- | --- | --- | --- | --- | --- | --- |
| Amare 2021^1^ | Ethiopia | Delphi study | Medicine - Surgery | 32 | Yes | Yes | No | Yes | No | Collaborate clinicians & educators | No | Yes |
| Baghus 2021^2^ | Netherlands | Delphi study | Medicine - Cross-specialty | 1 | No | Yes | Yes | Yes | No | Collaborate clinicians & educators | Yes | Yes |
| Bahji 2021^3^ | Canada | Systematic review | Medicine - Psychiatry | 6 | Yes | No | No | No | No | Not clearly reported | Yes | Yes |
| Benstead 2021^4^ | Europe | Case report | Medicine - Oncology | 3 | Yes | Yes | Yes | No | No | Collaborate clinicians & educators | Yes | Yes |
| Bonnie 2021^5^ | Netherlands | Cohort study | Medicine - GP | 6 | Yes | Yes | No | No | Yes | Not clearly reported | Yes | Yes |
| Cheung 2021^6^ | Canada | Cohort study | Medicine - Radiology | 3 | Yes | Yes | Yes | No | No | Not clearly reported | Yes | Yes |
| Datta 2021^7^ | India | Case report | Medicine - Sports/MSK/Orthopaedics | 114 | Yes | Yes | No | No | No | Collaborate clinicians & educators | Unclear | Yes |
| Datta 2021^8^ | India | Case report | Medicine - ENT | 335 | No | Yes | Yes | No | No | Collaborate clinicians & educators | Unclear | Yes |
| Dehghani Poudeh 2021^9^ | Other: Iran | Delphi study | Medicine - Internal | 28 | No | No | Yes | No | No | Collaborate clinicians & educators | Yes | Yes |
| Hennus 2021^10^ | Netherlands | Delphi study | Medicine - Paediatrics | 9 | Yes | No | Yes | No | No | Collaborate clinicians & educators | Yes | Yes |
| Hung 2021^11^ | United States | Commentary review | Medicine - Psychiatry | 13 | Yes | No | Yes | No | No | Not clearly reported | Yes | Yes |
| Kamp 2021^12^ | Germany | Consensus building approach | Medicine - Surgery Neuro-Oncology | 8 | No | No | Yes | No | No | Collaborate clinicians & educators | Yes | Yes |
| Karpinski 2021^13^ | Canada | Commentary review | Medicine - Cross-specialty / within scope of practice/training | 1 | Yes | No | Yes | No | No | Not clearly reported | Yes | Yes |
| Keating 2021^14^ | United States | Commentary review | Nursing / Nurse Practitioner | 2 | Yes | Yes | No | Yes | No | Not clearly reported | Yes | Yes |
| Khlevner 2021^15^ | United States | Professional guideline document | Medicine - Paediatrics | 1 | Yes | No | Yes | No | No | Not clearly reported | Yes | Yes |
| Kouzmina 2021^16^ | Canada | Evaluation | Medicine - Surgery | 6 | Yes | Yes | No | No | No | Not clearly reported | Yes | Yes |
| Kwan 2021^17^ | Canada | Case report | Medicine - Radiology | 8 | Yes | No | Yes | No | No | Collaborate clinicians & educators | Yes | Yes |
| Lindeman 2021^18^ | United States | Case report | Medicine - Surgery | 5 | Yes | Yes | No | No | No | Not clearly reported | Yes | Yes |
| Mishra 2021^19^ | Canada | Case report | Medicine - Radiology | 4 | Yes | Yes | Yes | No | No | Not clearly reported | Yes | Yes |
| Neumann 2021^20^ | Australia | Delphi study | Physiotherapy/Physical Therapy | 6 | No | Yes | Yes | No | Yes | Collaborate clinicians & educators | No | Yes |
| Osborn 2021^21^ | United States | Cohort study | Medicine - Sports/MSK/Orthopaedics | 7 | Yes | Yes | Yes | No | No | Not clearly reported | No | Yes |
| Rabski 2021^22^ | Canada | Qualitative research | Medicine - Neurosurgery | 15 | Yes | Yes | No | Yes | Yes | Not clearly reported | Yes | Yes |
| Safavi 2021^23^ | Canada | Conference abstract | Medicine - Radiation Oncology | 1 | Yes | Yes | No | Yes | No | Not clearly reported | No | Yes |
| Schwartz 2021^24^ | United States | Qualitative research | Medicine - Paediatrics | 24 | Yes | No | No | No | No | Other | Yes | Yes |
| Sebok-Syer 2021^25^ | United States | Commentary review | Medicine - Psychiatry | 5 | No | Yes | No | No | No | Not clearly reported | Yes | Unclear |
| Stahl 2021^26^ | United States | Case report | Medicine - Surgery | 5 | Yes | No | No | No | No | Not clearly reported | Yes | Yes |
| Stankovic 2021^27^ | Other: Italy | Professional guideline document | Medicine - Cross-specialty | 18 | Yes | No | No | No | No | Not clearly reported | No | Yes |
| Tanaka 2021^28^ | Japan | Delphi study | Medicine - Nephrology | 8 | Yes | No | No | No | No | Collaborate clinicians & educators | Yes | Yes |
| Turner 2021^29^ | United States | Qualitative research | Medicine - Paediatrics | 7 | Yes | No | No | Yes | No | Collaborate clinicians & educators | Yes | Yes |
| Watson 2021^30^ | Canada | Delphi study | Medicine - Sports/MSK/Orthopaedics | 49 | No | No | No | No | No | Collaborate clinicians & educators | Yes | Yes |
| Albright 2020^31^ | United States | Cohort study | Medicine - Surgery | 3 | Yes | No | Yes | No | Yes | Not clearly reported | Yes | Yes |
| Alismail 2020^32^ | United States | Commentary review | Respiratory Therapist | 8 | Yes | No | Yes | No | No | Not clearly reported | Yes | Yes |
| Andler 2020^33^ | United States | Cohort study | Medicine - Paediatrics | 2 | Yes | No | No | Yes | No | Not clearly reported | Yes | Yes |
| Anthamatten 2020^34^ | United States | Commentary review | Nursing / Nurse Practitioner | 4 | Yes | Yes | No | Yes | No | Not clearly reported | Yes | Yes |
| Ashokka 2020^35^ | Singapore | Commentary review | Medicine - Cross-specialty / within scope of practice/training | 1 | No | Yes | Yes | No | No | Collaborate clinicians & educators | Yes | Yes |
| Baer 2020^36^ | United States | Qualitative research | Medicine - Physical Medicine & Rehabilitation | 6 | Yes | Yes | No | No | Yes | Collaborate clinicians & educators | Unclear | Yes |
| Beamer 2020^37^ | United States | Case report | Nursing / Nurse Practitioner | 1 | No | Yes | No | No | No | Not clearly reported | Yes | Yes |
| Bokor 2020^38^ | United States; Canada | Conference abstract | Medicine - Adolescent | 1 | No | No | Yes | No | No | Not clearly reported | Yes | Yes |
| Chen 2020^39^ | United States | Evaluation | Medicine - Surgery | 6 | Yes | Yes | No | No | Yes | Not clearly reported | Unclear | Yes |
| Cohen 2020^40^ | United States | Delphi study | Medicine - Gastroenterology | 29 | Yes | Yes | Yes | No | No | Collaborate clinicians & educators | Yes | Yes |
| Cohen 2020^41^ | United States | Commentary review | Medicine - Gastroenterology - Advanced inflammatory bowel disease | 10 | Yes | Yes | Yes | No | No | Collaborate clinicians & educators | Yes | Yes |
| Crannell 2020^42^ | United States | Qualitative research | Medicine - Intensive care | 1 | No | Yes | Yes | No | No | Collaborate clinicians & educators | Yes | Yes |
| Dunne 2020^43^ | United States | Case report | Medicine - Internal | 12 | Yes | No | Yes | No | Yes | Collaborate clinicians & educators | Yes | Yes |
| Foong 2020^44^ | Singapore | Commentary review | Medicine - Surgery | 9 | Yes | Yes | Yes | Yes | No | Collaborate clinicians & educators | Yes | Yes |
| Gupta 2020^45^ | United States | Qualitative research | Medicine - Surgery | 5 | Yes | No | No | No | Yes | Not clearly reported | Yes | Yes |
| Hawkins 2020^46^ | United States | Commentary review | Dentistry | 10 | Yes | Yes | Yes | No | No | Collaborate clinicians & educators | Yes | Yes |
| Henry 2020^47^ | United States | Conference abstract | Medicine - Paediatrics | 11 | Yes | Yes | No | No | No | Collaborate clinicians & educators | Yes | Yes |
| Johnson 2020^48^ | United States | Cohort study | Medicine - Obs/gynae | 13 | Yes | Yes | No | Yes | No | Not clearly reported | Yes | Yes |
| Lum 2020^49^ | United States | Case report | Medicine - Telehealth | 1 | Yes | Yes | Yes | Yes | No | Collaborate clinicians & educators | Yes | Yes |
| Marty 2020^50^ | Other: Switzerland | Working group | Medicine - Anaesthetics/anaesthesiology | 16 | Yes | Yes | No | No | Yes | Collaborate clinicians & educators | Yes | Yes |
| Mink 2020^51^ | United States | Qualitative research | Medicine - Paediatrics | 7 | Yes | Yes | No | No | No | Not clearly reported | Yes | Yes |
| Moll-Khosrawi 2020^52^ | Germany | Delphi study | Medicine - Anaesthetics/anaesthesiology | 39 | Yes | Yes | No | No | No | Collaborate clinicians & educators | Yes | Yes |
| Naik 2020^53^ | United States | Case report | Medicine - Paediatrics | 1 | No | Yes | No | No | No | Not clearly reported | No | Yes |
| Pinilla 2020^54^ | Other: Switzerland | Systematic review | Medicine - Psychiatry | 4 | No | Yes | No | Yes | No | Clinicians | No | Yes |
| Prudhomme 2020^55^ | Canada | Qualitative research | Medicine - Emergency Medicine | 1 | Yes | Yes | No | No | Yes | Not clearly reported | Yes | Yes |
| Robson 2020^56^ | United States | Case report | Medicine - Paediatric Gastroenterology, Hepatology & Nutrition | 5 | Yes | No | No | No | Yes | Not clearly reported | Yes | Yes |
| Sauer 2020^57^ | United States | Professional guideline document | Medicine - Pediatric Gastroenterology, Hepatology and Nutrition | 27 | Yes | Yes | No | No | No | Collaborate clinicians & educators | Yes | Yes |
| Schumacher 2020^58^ | United States | Conference abstract | Medicine - Paediatrics | 1 | No | No | No | Yes | No | Not clearly reported | Yes | Yes |
| Schumacher 2020^59^ | United States | Case report | Medicine - Paediatrics | 17 | Yes | Yes | No | No | No | Collaborate clinicians & educators | Yes | Yes |
| Schumacher 2020^60^ | United States | Qualitative research | Medicine - Paediatrics | 17 | Yes | Yes | No | No | No | Collaborate clinicians & educators | Yes | Yes |
| Schwartz 2020^61^ | United States | Qualitative research | Medicine - Paediatrics | 2 | Yes | No | Yes | No | No | Not clearly reported | Yes | Yes |
| Sherbino 2020^62^ | Canada | Professional guideline document | Medicine - Emergency Medicine | 36 | Yes | Yes | Yes | No | No | Collaborate clinicians & educators | Yes | Yes |
| Solymos 2020^63^ | Ireland | Case report | Medicine - Anaesthetics/anaesthesiology | 4 | Yes | Yes | Yes | No | Yes | Collaborate clinicians & educators | Yes | Yes |
| Soran 2020^64^ | United States | Delphi study | Medicine - Internal | 16 | No | No | No | No | No | Collaborate clinicians & educators | Yes | Yes |
| Stahl 2020^65^ | United States | Case report | Medicine - Surgery | 5 | Yes | No | No | No | No | Not clearly reported | Yes | Yes |
| Stahl 2020^66^ | United States | Qualitative research | Medicine - Surgery | 5 | Yes | No | No | No | Yes | Not clearly reported | Yes | Unclear |
| Stahl 2020^67^ | United States | Commentary review | Medicine - Surgery | 5 | Yes | No | No | No | No | Not clearly reported | Yes | Yes |
| Stucke 2020^68^ | United States | Qualitative research | Medicine - Surgery | 1 | Yes | No | Yes | No | Yes | Not clearly reported | Yes | Yes |
| Tanner 2020^69^ | Europe | Professional guideline document | Medicine - Cardiology | 62 | Yes | Yes | Yes | No | No | Collaborate clinicians & educators | Yes | Yes |
| Thoma 2020^70^ | Canada | Cohort study | Medicine - Emergency Medicine | 28 | Yes | Yes | No | No | Yes | Not clearly reported | Yes | Yes |
| Benstead 2019^71^ | Europe | Professional guideline document | Medicine - Oncology | 14 | Yes | Yes | Yes | No | No | Collaborate clinicians & educators | Yes | Yes |
| Bonnie 2019^72^ | Netherlands | Qualitative research | Medicine - GP | 1 | Yes | Yes | Yes | No | Yes | Not clearly reported | Yes | Yes |
| Brasel 2019^73^ | United States | Commentary review | Medicine - Surgery | 5 | Yes | No | No | No | No | Collaborate clinicians & educators | Yes | Yes |
| Bratberg 2019^74^ | United States | Commentary review | Pharmacy/Pharmacology | 20 | Yes | No | Yes | No | No | Not clearly reported | Yes | Yes |
| Costello 2019^75^ | Canada | Conference abstract | Medicine - Emergency Medicine | 1 | Yes | No | No | No | No | Not clearly reported | Yes | Yes |
| Emke 2019^76^ | United States | Mixed methods | Medicine - Paediatrics | 3 | Yes | No | Yes | No | Yes | Collaborate clinicians & educators | Yes | Yes |
| Goodell 2019^77^ | United States | Scoping review | Medicine - Primary Care - Oral Health | 7 | Yes | Yes | Yes | No | No | Collaborate clinicians & educators | Yes | Yes |
| Greenberg 2019^78^ | United States | Commentary review | Medicine - Surgery | 5 | Yes | No | No | No | No | Not clearly reported | Yes | Yes |
| Gubitz 2019^79^ | Canada | Conference abstract | Medicine - Stroke | 6 | Yes | Yes | No | No | Yes | Collaborate clinicians & educators | Yes | Yes |
| Hart 2019^80^ | United States | Consensus working group | Medicine - Emergency Medicine | 11 | Yes | No | Yes | No | No | Collaborate clinicians & educators | Yes | Yes |
| Johnston 2019^81^ | United States | Delphi study | Medicine - Cross-specialty / within scope of practice/training | 1 | No | Yes | No | No | No | Not clearly reported | Unclear | Yes |
| Kane 2019^82^ | United States | Case report | Medicine - Paediatrics | 1 | Yes | Yes | No | No | No | Not clearly reported | Unclear | Yes |
| Karthikeyan 2019^83^ | India | Case report | Medicine - Otorhinolaryngology | 46 | Yes | Yes | No | Yes | No | Collaborate clinicians & educators | Yes | Yes |
| Malter 2019^84^ | United States | Commentary review | Medicine - Gastroenterology (GI) - Inflammatory Bowel Disease (IBD) | 8 | Yes | No | No | No | No | Collaborate clinicians & educators | Yes | Yes |
| Posel 2019^85^ | Canada | Cohort study | Medicine - Surgery | 3 | No | Yes | Yes | Yes | No | Clinicians | No | Yes |
| Sheth 2019^86^ | United States | Delphi study | Medicine - Radiology | 13 | Yes | No | No | No | No | Collaborate clinicians & educators | Yes | Yes |
| Shrivastava 2019^87^ | Nepal | Case report | Medicine - Community | 3 | No | Yes | No | No | No | Not clearly reported | Yes | Yes |
| Smit 2019^88^ | Netherlands | Case report | Medicine - Paediatrics | 9 | Yes | No | No | No | Yes | Collaborate clinicians & educators | Yes | Yes |
| Surjadi 2019^89^ | United States | Commentary review | Nursing / Nurse Practitioner | 1 | Yes | No | Yes | Yes | No | Collaborate clinicians & educators | Yes | Yes |
| Valentine 2019^90^ | Australia | Cohort study | Medicine - GP | 13 | Yes | No | No | Yes | No | Collaborate clinicians & educators | Yes | Yes |
| vanBockel 2019^91^ | Netherlands | Case report | Medicine - Intensive care | 15 | Yes | No | Yes | No | No | Collaborate clinicians & educators | Yes | Yes |
| Wang 2019^92^ | United States | Commentary review | Medicine - Internal | 3 | Yes | Yes | No | Yes | No | Not clearly reported | Yes | Yes |
| Westein 2019^93^ | Netherlands | Case report | Pharmacy/Pharmacology | 36 | Yes | Yes | No | Yes | No | Collaborate clinicians & educators | Yes | Yes |
| Aggarwal 2018^94^ | Canada | Delphi study | Medicine - Obs/gynae | 15 | Yes | No | No | No | Yes | Clinicians | Yes | Yes |
| Auble 2018^95^ | United States | Conference abstract | Medicine - Paediatrics | 2 | Yes | No | Yes | No | No | Not clearly reported | Yes | Yes |
| Barakat 2018^96^ | United States | Commentary review | Medicine - Internal | 12 | Yes | Yes | Yes | No | No | Collaborate clinicians & educators | Yes | Yes |
| Borman-Shoap 2018^97^ | United States | Conference abstract | Medicine - Emergency Medicine | 2 | No | No | No | No | No | Not clearly reported | Yes | Yes |
| Eckberg 2018^98^ | United States | Conference abstract | Medicine - Paediatrics | 1 | Yes | No | No | No | No | Not clearly reported | Yes | Yes |
| Emke 2018^99^ | United States | Case report | Medicine - Paediatrics | 1 | No | No | No | No | Yes | Not clearly reported | Yes | Yes |
| Hurd 2018^100^ | United States | Conference abstract | Medicine - Cross-specialty / within scope of practice/training | 2 | Yes | Yes | No | Yes | No | Not clearly reported | Yes | Yes |
| Ko 2018^101^ | Canada | Conference abstract | Medicine - Oncology | 2 | Yes | Yes | No | Yes | No | Not clearly reported | Unclear | Yes |
| Kovatch 2018^102^ | United States | Case report | Medicine - Surgery | 4 | Yes | No | Yes | No | No | Collaborate clinicians & educators | Yes | Yes |
| McMillan 2018^103^ | United States | Commentary, review, letter or opinion | Medicine - Paediatrics | 1 | Yes | No | Yes | No | No | Not clearly reported | Yes | Yes |
| Mink 2018^104^ | United States | Delphi study | Medicine - Paediatrics | 6 | Yes | Yes | No | Yes | No | Collaborate clinicians & educators | Yes | Yes |
| Pinsk 2018^105^ | Canada | Commentary review | Medicine - Other post-qualification specialty | 39 | Yes | Yes | No | No | Yes | Clinicians | No | Yes |
| Santhosh 2018^106^ | United States | Conference abstract | Medicine - Pulmonary & critical care medicine | 2 | Yes | No | No | No | No | Not clearly reported | Unclear | Yes |
| Schnobrich 2018^107^ | United States | Case report | Medicine - Internal | 1 | No | Yes | Yes | No | No | Not clearly reported | Yes | Yes |
| Soran 2018^108^ | United States | Case report | Medicine - Internal | 14 | No | No | No | No | No | Collaborate clinicians & educators | Yes | Yes |
| Steiman 2018^109^ | United States | Qualitative research | Medicine - Surgery | 2 | Yes | No | No | No | No | Collaborate clinicians & educators | No | Yes |
| Taylor 2018^110^ | Canada | Delphi study | Medicine - Internal | 29 | Yes | No | No | Yes | No | Collaborate clinicians & educators | Yes | Yes |
| Wagner 2018^111^ | United States | Commentary review | Nursing / Nurse Practitioner | 1 | Yes | Yes | Yes | Yes | No | Collaborate clinicians & educators | Yes | Yes |
| Barratt 2017^112^ | United States | Conference abstract | Medicine Neurology | 4 | Yes | Yes | No | Yes | Yes | Not clearly reported | Yes | Yes |
| Fehr 2017^113^ | Germany | Delphi study | Medicine - Paediatrics | 12 | Yes | Yes | Yes | No | No | Collaborate clinicians & educators | Yes | Yes |
| Fix 2017^114^ | United States | Conference abstract | Medicine - Gastroenterology (GI)/transplant hepatology (TH) | 6 | No | Yes | No | No | No | Collaborate clinicians & educators | Yes | Yes |
| Kumar 2017^115^ | India | Case report | Medicine - Pathology | 1 | No | Yes | Yes | No | No | Not clearly reported | Yes | Yes |
| Landzaat 2017^116^ | United States | Case report | Medicine - Hospice and Palliative | 17 | Yes | No | Yes | No | No | Collaborate clinicians & educators | Yes | Yes |
| Mallow 2017^117^ | United States | Delphi study | Medicine - Physical Medicine and Rehabilitation (PM&R) | 19 | Yes | Yes | No | No | No | Collaborate clinicians & educators | Yes | Yes |
| McMurray 2017^118^ | Canada | Qualitative research | Medicine - Resuscitation | 1 | Yes | No | Yes | No | No | Not clearly reported | Yes | Yes |
| Moloughney 2017^119^ | Canada | Case report | Medicine - Public Health & Preventative Medicine (PHPM) | 20 | No | Yes | Yes | No | No | Collaborate clinicians & educators | Yes | Yes |
| Moore 2017^120^ | Australia | Case report | Medicine - Surgery | 13 | Yes | Yes | Yes | No | No | Not clearly reported | Yes | Yes |
| Parker 2017^121^ | United States | Delphi study | Medicine - Other post-qualification specialty | 13 | No | Yes | No | No | No | Educators | No | Yes |
| Powell 2017^122^ | United States | Commentary review | Medicine - Paediatrics | 17 | Yes | Yes | No | No | No | Not clearly reported | Yes | Yes |
| Srivastava 2017^123^ | United States | Case report | Medicine - Paediatrics | 6 | Yes | No | Yes | No | No | Collaborate clinicians & educators | Yes | Yes |
| TenCate 2017^124^ | Multi-national - Netherlands, USA, Australia | Commentary review | Medicine - Obs/gynae | 7 | No | No | Yes | No | No | Not clearly reported | Yes | Yes |
| Williams 2017^125^ | United States | Case report | Medicine - Paediatrics | 1 | No | Yes | No | Yes | Yes | Not clearly reported | No | Yes |
| Brown 2016^126^ | United States | Case report | Medicine - Rheumatology | 14 | Yes | No | Yes | No | No | Collaborate clinicians & educators | Yes | Yes |
| Chaplin 2016^127^ | United States | Conference abstract | Medicine - Cross-specialty | 1 | Yes | Yes | No | No | No | Not clearly reported | Yes | Yes |
| Cummings 2016^128^ | United States | Commentary review | Medicine - Paediatrics | 12 | Yes | No | Yes | No | No | Not clearly reported | Yes | Yes |
| Deitte 2016^129^ | United States | Case report | Medicine - Radiology | 10 | Yes | No | Yes | Yes | No | Collaborate clinicians & educators | Yes | Yes |
| Dwyer 2016^130^ | Canada | Cohort study | Medicine - Cross-specialty | 10 | Yes | No | Yes | No | No | Not clearly reported | Yes | Yes |
| Kwan 2016^131^ | Australia | Case report | Medicine - Emergency Medicine | 2 | Yes | Yes | Yes | No | No | Collaborate clinicians & educators | Yes | Yes |
| Liu 2016^132^ | Australia | Conference abstract | Medicine - Urology | 4 | No | Yes | No | No | No | Not clearly reported | Yes | Yes |
| Manders 2016^133^ | United States | Meeting abstract | Medicine - Paediatrics | 1 | Yes | No | No | No | No | Collaborate clinicians & educators | Yes | Yes |
| Meade 2016^134^ | United States | Case report | Medicine - Internal | 1 | Yes | No | Yes | No | No | Collaborate clinicians & educators | Yes | Yes |
| Menard-Katcher 2016^135^ | United States | Conference abstract | Medicine - Internal | 1 | No | No | No | No | No | Not clearly reported | Yes | Yes |
| Post 2016^136^ | United States | Qualitative research | Medicine - Internal | 229 | Yes | No | No | No | Yes | Not clearly reported | Yes | Yes |
| Touchie 2016^137^ | Canada | Commentary review | Medicine - Cross-specialty | 2 | No | Yes | No | No | No | Not clearly reported | Yes | Yes |
| vanHouwelingen 2016^138^ | Netherlands | Delphi study | Nursing / Nurse Practitioner | 14 | No | No | Yes | Yes | No | Collaborate clinicians & educators | Yes | Yes |
| Weiss 2016^139^ | United States | Case report | Medicine - Psychiatry | 3 | No | Yes | Yes | No | Yes | Collaborate clinicians & educators | Yes | Yes |
| Wisman-Zwarter 2016^140^ | Netherlands | Delphi study | Medicine - Anaesthetics/anaesthesiology | 45 | Yes | No | Yes | No | No | Not clearly reported | Yes | Yes |
| Wolfel 2016^141^ | Germany | Qualitative research | Medicine - Cross-specialty | 1 | No | Yes | Yes | No | No | Collaborate clinicians & educators | Yes | Yes |
| Adams 2015^142^ | Australia | Commentary review | Medicine - Psychiatry | 1 | No | No | No | No | No | Not clearly reported | Unclear | Yes |
| Block 2015^143^ | United States | Cohort study | Medicine - Internal | 25 | Yes | No | Yes | No | No | Collaborate clinicians & educators | Yes | Yes |
| Clardy 2015^144^ | United States | Commentary review | Medicine - pulmonary / critical care | 3 | Yes | No | Yes | No | No | Not clearly reported | Yes | Yes |
| Dilly 2015^145^ | United States | Conference abstract | Medicine - GI | 1 | Yes | No | Yes | No | No | Not clearly reported | Yes | Yes |
| Hamburger 2015^146^ | Canada | Qualitative research | Medicine - Paediatrics | 1 | Yes | No | Yes | No | Yes | Collaborate clinicians & educators | Yes | Yes |
| Meade 2015^147^ | United States | Case report | Medicine - Internal | 1 | Yes | No | Yes | No | No | Not clearly reported | Yes | Yes |
| Myers 2015^148^ | Canada | Case report | Medicine - Palliative | 12 | No | Yes | Yes | No | No | Collaborate clinicians & educators | Yes | Yes |
| Santhosh 2015^149^ | United States | Conference abstract | Medicine - Internal | 1 | Yes | No | No | No | No | Not clearly reported | Unclear | Yes |
| Schultz 2015^150^ | Canada | Commentary review | Medicine - Family medicine | 44 | Yes | No | Yes | No | No | Collaborate clinicians & educators | Yes | Yes |
| Yuan 2015^151^ | United States | Commentary review | Medicine - Nephrology | 5 | Yes | No | Yes | No | No | Collaborate clinicians & educators | Yes | Yes |
| Beeson 2014^152^ | United States | Conference abstract | Medicine - Emergency Medicine | 1 | No | No | Yes | No | No | Not clearly reported | Yes | Yes |
| Beeson 2014^153^ | United States | Commentary review | Medicine - Emergency Medicine | 1 | Yes | No | Yes | No | No | Collaborate clinicians & educators | Yes | Yes |
| Chan 2014^154^ | United States | Case report | Medicine - Internal | 1 | Yes | No | Yes | Yes | No | Collaborate clinicians & educators | Yes | Yes |
| Klein 2014^155^ | United States | Commentary review | Medicine - Paediatrics | 1 | Yes | No | Yes | No | No | Collaborate clinicians & educators | Yes | Yes |
| Leipzig 2014^156^ | United States | Case report | Medicine - Geriatrics | 12 | Yes | No | Yes | Yes | No | Collaborate clinicians & educators | Yes | Unclear |
| Ng 2014^157^ | Singapore | Case report | Medicine - Cross-specialty | 5 | No | No | No | No | No | Not clearly reported | Yes | Yes |
| O'Keeffe 2014^158^ | Australia | Commentary review | Medicine - Paediatrics | 14 | No | No | Yes | No | No | Clinicians | No | Yes |
| Oversight Working 2014^159^ | United States | Other | Medicine - Other post-qualification specialty | 13 | Yes | No | Yes | No | No | Collaborate clinicians & educators | No | Yes |
| Rose 2014^160^ | United States | Case report | Medicine - Gastroenterology | 13 | Yes | No | Yes | No | Yes | Collaborate clinicians & educators | Yes | Yes |
| Shaughnessy 2014^161^ | UK | Case report | Medicine - Family Medicine | 1 | No | No | Yes | No | No | Not clearly reported | Yes | Yes |
| Touchie 2014^162^ | United States | Case report | Medicine - Cross-specialty | 10 | No | Yes | No | No | No | Collaborate clinicians & educators | Yes | Yes |
| Trevallion 2014^163^ | Australia | Conference abstract | Medicine - Psychiatry | 2 | Yes | Yes | No | Yes | No | Not clearly reported | Yes | Yes |
| vanLoon 2014^164^ | Netherlands | Commentary review | Medicine - Cross-specialty | 7 | No | No | No | No | No | Not clearly reported | Yes | Yes |
| Yuan 2014^165^ | United States | Cohort study | Medicine - Other post-qualification specialty | 1 | Yes | No | Yes | No | Yes | Collaborate clinicians & educators | No | Yes |
| Berberat 2013^166^ | Germany | Commentary review | Medicine - Cross-specialty | 8 | Yes | Yes | Yes | No | Yes | Not clearly reported | Yes | Yes |
| Chang 2013^167^ | United States | Case report | Medicine - Internal | 25 | Yes | No | Yes | Yes | No | Collaborate clinicians & educators | Yes | Yes |
| Hauer 2013^168^ | United States | Delphi study | Medicine - Internal | 30 | Yes | Yes | No | No | Yes | Collaborate clinicians & educators | Yes | Yes |
| Hauer 2013^169^ | United States | Evaluation | Medicine - Internal | 2 | Yes | No | Yes | No | Yes | Not clearly reported | Yes | Yes |
| Shaughnessy 2013^170^ | UK | Delphi study | Medicine - Family Medicine | 76 | No | Yes | No | No | No | Collaborate clinicians & educators | Yes | Yes |
| tenCate 2012^171^ | United States | Commentary review | Medicine - Cross-specialty | 1 | Yes | Yes | Yes | Yes | No | Collaborate clinicians & educators | Yes | Yes |
| Boyce 2011^172^ | Other: Australia and New Zealand | Qualitative research | Medicine - Psychiatry | 4 | Yes | Yes | Yes | Yes | No | Collaborate clinicians & educators | Yes | Unclear |
| tenCate 2007^173^ | Netherlands | Commentary review | Medicine - Obs/gynae | 18 | Yes | No | Yes | No | No | Not clearly reported | Yes | Yes |

**REFERENCES**

1. Amare EM, Siebeck M, Sendekie TY, Fischer MR, Berndt M. Development of an Entrustable Professional Activities (EPA) Framework to Inform Surgical Residency Training Programs in Ethiopia: A Three-round National Delphi Method Study. *Journal of Surgical Education*. 2021;19:19. doi:https://dx.doi.org/10.1016/j.jsurg.2021.06.023

2. Baghus A, Giroldi E, Muris J, et al. Identifying Entrustable Professional Activities for Shared Decision Making in Postgraduate Medical Education: A National Delphi Study. *Academic Medicine*. 2021;96(1):126-133. doi:https://dx.doi.org/10.1097/ACM.0000000000003618

3. Bahji A, Smith J, Danilewitz M, Crockford D, El-Guebaly N, Stuart H. Towards competency-based medical education in addictions psychiatry: a systematic review. *Canadian Medical Education Journal [Electronic Resource]*. 2021;12(3):126-141. doi:https://dx.doi.org/10.36834/cmej.69739

4. Benstead K, Lara PC, Eller Y, et al. Clinical oncology module for the ESTRO core curriculum. *Radiotherapy & Oncology*. 2021;156:19-22. doi:https://dx.doi.org/10.1016/j.radonc.2020.11.029

5. Bonnie LHA, Nasori M, Visser MRM, Kramer AWM, van Dijk N. Feasibility, and validity aspects of Entrustable Professional Activity (EPA)-based assessment in general practice training. *Education for Primary Care*. 2021:1-8. doi:https://dx.doi.org/10.1080/14739879.2021.1951127

6. Cheung K, Rogoza C, Chung AD, Kwan BYM. Analyzing the Administrative Burden of Competency Based Medical Education. *Canadian Association of Radiologists Journal*. 2021;doi:http://dx.doi.org/10.1177/08465371211038963

7. Datta K, Guru CS, Krishnan A, Datta R, Sharma D. Entrustable Professional Activities (EPAs) and milestones for MD sports medicine: A proposed portfolio. *Medical journal, Armed Forces India*. 2021;77(Suppl 1):S129-S133. doi:10.1016/j.mjafi.2020.12.026

8. Datta R, Raghavan D, Anand V, et al. Identifying entrustable professional activities for post-graduation in ENT: What should an ENT specialist be able to do? *Medical journal, Armed Forces India*. 2021;77(Suppl 1):S168-S172. doi:10.1016/j.mjafi.2020.12.031

9. Dehghani Poudeh M, Mohammadi A, Mojtahedzadeh R, Yamani N. Entrustability levels of general internal medicine residents. *BMC Medical Education*. 2021;21(1):185. doi:https://dx.doi.org/10.1186/s12909-021-02624-9

10. Hennus MP, Nusmeier A, van Heesch GGM, et al. Development of entrustable professional activities for paediatric intensive care fellows: A national modified Delphi study. *PLoS ONE [Electronic Resource]*. 2021;16(3 March) (no pagination)doi:http://dx.doi.org/10.1371/journal.pone.0248565

11. Hung EK, Jibson M, Sadhu J, et al. Wresting with Implementation: a Step-By-Step Guide to Implementing Entrustable Professional Activities (EPAs) in Psychiatry Residency Programs. *Academic Psychiatry*. 2021;45(2):210-216. doi:https://dx.doi.org/10.1007/s40596-020-01341-7

12. Kamp MA, Malzkorn B, von Sass C, et al. Proposed definition of competencies for surgical neuro-oncology training. *Journal of Neuro-Oncology*. 2021;153(1):121-131. doi:http://dx.doi.org/10.1007/s11060-021-03750-6

13. Karpinski J, Frank JR. The Role of EPAs in Creating a National System of Time-Variable Competency-Based Medical Education. *Academic Medicine*. 2021;96(7S):S36-S41. doi:https://dx.doi.org/10.1097/ACM.0000000000004087

14. Keating S, McLeod-Sordjan R, Lemp M, Willenbrock D, Fried AM, Cassara M. Evaluating Entrustable Professional Activities in a Nurse Practitioner Readiness for Practice Simulation. *Journal for Nurse Practitioners*. 2021;17(5):611-614. doi:10.1016/j.nurpra.2021.01.003

15. Khlevner J, Rosen R, Ambartsumyan L, et al. Development of Entrustable Professional Activities and Standards in Training in Pediatric Neurogastroenterology and Motility: North American Society for Pediatric Gastroenterology, Hepatology and Nutrition and American Neurogastroenterology and Motility Society Position Paper. *Journal of Pediatric Gastroenterology & Nutrition*. 2021;72(1):168-180. doi:https://dx.doi.org/10.1097/MPG.0000000000002965

16. Kouzmina E, Mann S, Chaplin T, Zevin B. An Evaluation of the Surgical Foundations Curriculum: A National Study. *Journal of Surgical Education*. 2021;78(3):914-926. doi:https://dx.doi.org/10.1016/j.jsurg.2020.10.002

17. Kwan BYM, Mbanwi A, Cofie N, et al. Creating a Competency-Based Medical Education Curriculum for Canadian Diagnostic Radiology Residency (Queen's Fundamental Innovations in Residency Education)-Part 1: Transition to Discipline and Foundation of Discipline Stages. *Canadian Association of Radiologists Journal*. 2021;72(3):372-380. doi:https://dx.doi.org/10.1177/0846537119894723

18. Lindeman B, Brasel K, Minter RM, Buyske J, Grambau M, Sarosi G. A Phased Approach: The General Surgery Experience Adopting Entrustable Professional Activities in the United States. *Academic Medicine*. 2021;96(7S):S9-S13. doi:https://dx.doi.org/10.1097/ACM.0000000000004107

19. Mishra S, Chung A, Rogoza C, et al. Creating a Competency-Based Medical Education Curriculum for Canadian Diagnostic Radiology Residency (Queen's Fundamental Innovations in Residency Education)-Part 2: Core of Discipline Stage. *Canadian Association of Radiologists Journal*. 2021;doi:http://dx.doi.org/10.1177/0846537121993058

20. Neumann PB, Radi N, Gerdis TL, et al. Development of a multinational, multidisciplinary competency framework for physiotherapy training in pessary management: an E-Delphi study. *International Urogynecology Journal*. 2021;05:05. doi:https://dx.doi.org/10.1007/s00192-021-04843-6

21. Osborn PM, Dowd TC, Schmitz MR, Lybeck DO. Establishing an Orthopedic Program-Specific, Comprehensive Competency-Based Education Program. *Journal of Surgical Research*. 2021;259:399-406. doi:https://dx.doi.org/10.1016/j.jss.2020.09.016

22. Rabski JE, Saha A, Cusimano MD. Setting standards of performance expected in neurosurgery residency: A study on entrustable professional activities in competency-based medical education. *American Journal of Surgery*. 2021;221(2):388-393. doi:https://dx.doi.org/10.1016/j.amjsurg.2020.12.014

23. Safavi AH, Sienna J, Strang BK, Hann C. Competency-Based Medical Education in Canadian Radiation Oncology Residency Training: An Institutional Implementation Pilot Study. *International Journal of Radiation Oncology Biology Physics*. 2021;111(1):e21. doi:http://dx.doi.org/10.1016/j.ijrobp.2021.05.176

24. Schwartz A, Borman-Shoap E, Carraccio C, et al. Learner Levels of Supervision Across the Continuum of Pediatrics Training. *Academic Medicine*. 2021;96(7S):S42-S49. doi:https://dx.doi.org/10.1097/ACM.0000000000004095

25. Sebok-Syer SS, Gingerich A, Holmboe ES, Lingard L, Turner DA, Schumacher DJ. Distant and Hidden Figures: Foregrounding Patients in the Development, Content, and Implementation of Entrustable Professional Activities. *Academic Medicine*. 2021;96(7S):S76-S80. doi:https://dx.doi.org/10.1097/ACM.0000000000004094

26. Stahl CC, Jung SA, Rosser AA, et al. Natural language processing and entrustable professional activity text feedback in surgery: A machine learning model of resident autonomy. *American Journal of Surgery*. 2021;221(2):369-375. doi:https://dx.doi.org/10.1016/j.amjsurg.2020.11.044

27. Stankovic I, Muraru D, Fox K, et al. Level 1 of Entrustable Professional Activities in adult echocardiography: a position statement from the EACVI regarding the training and competence requirements for selecting and interpreting echocardiographic examinations. *European heart journal Cardiovascular Imaging*. 2021;11doi:http://dx.doi.org/10.1093/ehjci/jeab143

28. Tanaka A, Kondo T, Urushibara-Miyachi Y, Maruyama S, Nishigori H. Development of entrustable professional activities for residents rotating nephrology department in a Japanese university hospital: a Delphi study. *BMJ Open*. 2021;11(8):e047923. doi:https://dx.doi.org/10.1136/bmjopen-2020-047923

29. Turner DA, Schwartz A, Carraccio C, et al. Continued Supervision for the Common Pediatric Subspecialty Entrustable Professional Activities May Be Needed Following Fellowship Graduation. *Academic medicine : journal of the Association of American Medical Colleges*. 2021;S. 96(7):S22-S28. doi:http://dx.doi.org/10.1097/ACM.0000000000004091

30. Watson A, Leroux T, Ogilvie-Harris D, et al. Entrustable Professional Activities in Orthopaedics. *JB & JS Open Access*. 2021;6(2):Apr-Jun. doi:https://dx.doi.org/10.2106/JBJS.OA.20.00010

31. Albright JB, Meier AH, Ruangvoravat L, VanderMeer TJ. Association Between Entrustable Professional Activities and Milestones Evaluations: Real-time Assessments Correlate With Semiannual Reviews. *Journal of Surgical Education*. 2020;77(6):e220-e228. doi:https://dx.doi.org/10.1016/j.jsurg.2020.07.027

32. Alismail A, Lopez D. Clinical Competencies in Advanced Practice Respiratory Therapy Education: Is It Time to Entrust the Learner? *Advances in Medical Education & Practice*. 2020;11:83-89. doi:https://dx.doi.org/10.2147/AMEP.S239376

33. Andler C, Daya S, Kowalek K, Boscardin C, van Schaik SM. E-ASSESS: Creating an EPA Assessment Tool for Structured Simulated Emergency Scenarios. *Journal of Graduate Medical Education*. 2020;12(2):153-158. doi:https://dx.doi.org/10.4300/JGME-D-19-00533.1

34. Anthamatten A, Pfieffer ML, Richmond A, Glassford M. Exploring the Utility of Entrustable Professional Activities as a Framework to Enhance Nurse Practitioner Education. *Nurse Educator*. 2020;45(2):83-87. doi:https://dx.doi.org/10.1097/NNE.0000000000000697

35. Ashokka B, Chakraborty A, Subramanian BJ, Karmakar MK, Chan V. Reconfiguring the scope and practice of regional anesthesia in a pandemic: The COVID-19 perspective. *Regional Anesthesia and Pain Medicine*. 2020;45(7):536-543. doi:http://dx.doi.org/10.1136/rapm-2020-101541

36. Baer HR, Gilbert AR, Forster JE, Ketchum NC, Mallow M, Nguyen VQC. Use of the Electrodiagnostic Entrustable Professional Activity for Competency Assessment in Physical Medicine and Rehabilitation Training Programs. *American Journal of Physical Medicine & Rehabilitation*. 2020;99(1):81-85. doi:https://dx.doi.org/10.1097/PHM.0000000000001302

37. Beamer JC, Kromer RS, Jeffery AD. Imagining an Orientation Built on Trust. *Journal for Nurses in Professional Development*. 2020;36(1):2-6. doi:https://dx.doi.org/10.1097/NND.0000000000000602

38. Bokor BR, Lemke M, Greenberg I, Coddington DA, Bhansali P. 39. Motivational Interviewing: The Current State of Teaching and Assessment in Adolescent Medicine Fellowships. *Academic pediatrics*. 2020;20(7):e19-e20. doi:http://dx.doi.org/10.1016/j.acap.2020.06.060

39. Chen XP, Harzman A, Cochran A, Ellison EC. Evaluation of an instrument to assess resident surgical entrustable professional activities (SEPAs). *American Journal of Surgery*. 2020;220(1):4-7. doi:https://dx.doi.org/10.1016/j.amjsurg.2019.08.026

40. Cohen BL, Gallinger ZR, Ha C, et al. Development of Entrustable Professional Activities for Advanced Inflammatory Bowel Disease Fellowship Training in the United States. *Inflammatory Bowel Diseases*. 2020;26(9):1291-1305. doi:https://dx.doi.org/10.1093/ibd/izaa177

41. Cohen BL, Hanauer SB. Executive Summary of 'Development of Entrustable Professional Activities for Advanced Inflammatory Bowel Disease Fellowship Training in the United States'. *American Journal of Gastroenterology*. 2020;115(9):1362-1366. doi:https://dx.doi.org/10.14309/ajg.0000000000000809

42. Crannell WC, Zakhary B, Hamilton H, Brasel K, Zonies D. Design of an entrustable professional activity for adult extracorporeal membrane oxygenation. *Surgery open science*. 2020;2(1):42-45. doi:10.1016/j.sopen.2019.09.001

43. Dunne D, Green M, Tetrault J, Barakat LA. Development of a Novel Competency-Based Evaluation System for HIV Primary Care Training: the HIV Entrustable Professional Activities. *Journal of General Internal Medicine*. 2020;35(1):331-335. doi:https://dx.doi.org/10.1007/s11606-019-04956-1

44. Foong TW, Tan JKH, Ashokka B, et al. How do we turn surgical residents into safe intensive care unit clinicians? An Entrustable Professional Activities guided framework. *British Journal of Surgery*. 2020;107(11):e491-e493. doi:https://dx.doi.org/10.1002/bjs.11949

45. Gupta A, Watkins AC, Fahey TJ, Barie PS, Narayan M. Entrustable Professional Activities: Do General Surgery Residents Trust Them? *Journal of Surgical Education*. 2020;77(3):520-526. doi:https://dx.doi.org/10.1016/j.jsurg.2019.12.005

46. Hawkins J, Heir G, Okeson J, Shaefer J. Entrustable Professional Activities in Postgraduate Orofacial Pain Programs. *Journal of Oral & Facial Pain and Headache*. 2020;34(3):255-264. doi:https://dx.doi.org/10.11607/ofph.2640

47. Henry D, Huth K, Fabersunne CC, Schumacher D, Shah N. 79. Demystifying Complex Care: Development of Entrustable Professional Activities in Pediatric Complex Care. *Academic pediatrics*. 2020;20(7):e37-e38. doi:http://dx.doi.org/10.1016/j.acap.2020.06.100

48. Johnson NR, Pelletier A, Berkowitz LR. Mini-Clinical Evaluation Exercise in the Era of Milestones and Entrustable Professional Activities in Obstetrics and Gynaecology: Resume or Reform? *Journal of Obstetrics & Gynaecology Canada: JOGC*. 2020;42(6):718-725. doi:https://dx.doi.org/10.1016/j.jogc.2019.10.002

49. Lum E, van Galen LS, Car J. Competency-Based Training for Entrustment in Telehealth Consultations. *Pediatric Clinics of North America*. 2020;67(4):735-757. doi:https://dx.doi.org/10.1016/j.pcl.2020.04.013

50. Marty AP, Schmelzer S, Thomasin RA, et al. Agreement between trainees and supervisors on first-year entrustable professional activities for anaesthesia training. *British Journal of Anaesthesia*. 2020;125(1):98-103. doi:https://dx.doi.org/10.1016/j.bja.2020.04.009

51. Mink R, Herman BE, Carraccio C, et al. Agreement of Program Directors With Clinical Competency Committees for Fellow Entrustment. *Journal of Medical Education & Curricular Development*. 2020;7:2382120520936613. doi:https://dx.doi.org/10.1177/2382120520936613

52. Moll-Khosrawi P, Ganzhorn A, Zollner C, Schulte-Uentrop L. Development and validation of a postgraduate anaesthesiology core curriculum based on Entrustable Professional Activities: a Delphi study. *GMS Journal for Medical Education*. 2020;37(5):Doc52. doi:https://dx.doi.org/10.3205/zma001345

53. Naik VV, Scheurer J, Madhok M, Vora S, Nelson S, Borman-Shoap E. 78. Performance under Pressure: The Use of Epas to Standardize Pediatric Mock Code Feedback. *Academic pediatrics*. 2020;20(7):e37. doi:http://dx.doi.org/10.1016/j.acap.2020.06.099

54. Pinilla S, Lenouvel E, Strik W, Kloppel S, Nissen C, Huwendiek S. Entrustable Professional Activities in Psychiatry: A Systematic Review. *Academic psychiatry : the journal of the American Association of Directors of Psychiatric Residency Training and the Association for Academic Psychiatry*. 2020;44(1):37-45. doi:http://dx.doi.org/10.1007/s40596-019-01142-7

55. Prudhomme N, O'Brien M, McConnell MM, Dudek N, Cheung WJ. Relationship between ratings of performance in the simulated and workplace environments among emergency medicine residents. *Canadian Journal of Emergency Medicine*. 2020;22(6):811-818. doi:http://dx.doi.org/10.1017/cem.2020.388

56. Robson J, Lusman SS, Lee CK, et al. Pediatric Gastroenterology, Hepatology, and Nutrition Entrustable Professional Activities: Development of an Assessment Tool and Curricular Resources. *Journal of Pediatric Gastroenterology and Nutrition*. 2020;71(1):e40-e45. doi:http://dx.doi.org/10.1097/MPG.0000000000002715

57. Sauer CG, Robson J, Turmelle YP, et al. North American Society for Pediatric Gastroenterology, Hepatology, and Nutrition Position Paper on Entrustable Professional Activities: Development of Pediatric Gastroenterology, Hepatology, and Nutrition Entrustable Professional Activities. *Journal of Pediatric Gastroenterology and Nutrition*. 2020;71(1):136-143. doi:http://dx.doi.org/10.1097/MPG.0000000000002684

58. Schumacher D, Schwartz A, Famiglietti H, et al. 73. Etiology of Unable to Assess Entrustable Professional Activities in a National Study. *Academic pediatrics*. 2020;20(7):e34-e35. doi:http://dx.doi.org/10.1016/j.acap.2020.06.094

59. Schumacher DJ, Schwartz A, Zenel JA, et al. Narrative Performance Level Assignments at Initial Entrustment and Graduation: Integrating EPAs and Milestones to Improve Learner Assessment. *Academic medicine : journal of the Association of American Medical Colleges*. 2020;95(11):1736-1744. doi:http://dx.doi.org/10.1097/ACM.0000000000003300

60. Schumacher DJ, West DC, Schwartz A, et al. Longitudinal Assessment of Resident Performance Using Entrustable Professional Activities. *JAMA Network Open*. 2020;3(1) (no pagination)doi:http://dx.doi.org/10.1001/jamanetworkopen.2019.19316

61. Schwartz A, Balmer DF, Borman-Shoap E, et al. Shared Mental Models Among Clinical Competency Committees in the Context of Time-Variable, Competency-Based Advancement to Residency. *Academic Medicine*. 2020;95(11S Association of American Medical Colleges Learn Serve Lead: Proceedings of the 59th Annual Research in Medical Education Presentations):S95-S102. doi:https://dx.doi.org/10.1097/ACM.0000000000003638

62. Sherbino J, Bandiera G, Doyle K, et al. The competency-based medical education evolution of Canadian emergency medicine specialist training. *CJEM Canadian Journal of Emergency Medical Care*. 2020;22(1):95-102. doi:https://dx.doi.org/10.1017/cem.2019.417

63. Solymos O, Snyman L, Condon E, Power C, Boland J. Moving beyond the technical skills and promoting professionalism-the experience of the College of Anaesthesiologists of Ireland with incorporating the Medical Council Eight Domains of Good Professional Practice into Entrustable Professional Activities. *Irish Journal of Medical Science*. 2020;189(4):1379-1389. doi:https://dx.doi.org/10.1007/s11845-020-02216-4

64. Soran C, Laponis R, Summerville S, et al. Identifying Entrustable Professional Activities for Internal Medicine Residents in Ambulatory Continuity Practice. *Journal of General Internal Medicine*. 2020;35(6):1917-1919. doi:http://dx.doi.org/10.1007/s11606-019-05430-8

65. Stahl CC, Collins E, Jung SA, et al. Implementation of Entrustable Professional Activities into a General Surgery Residency. *Journal of Surgical Education*. 2020;77(4):739-748. doi:http://dx.doi.org/10.1016/j.jsurg.2020.01.012

66. Stahl CC, Jung SA, Rosser AA, et al. Entrustable Professional Activities in General Surgery: Trends in Resident Self-Assessment. *Journal of Surgical Education*. 2020;77(6):1562-1567. doi:https://dx.doi.org/10.1016/j.jsurg.2020.05.005

67. Stahl CC, Minter RM. New Models of Surgical Training. *Advances in Surgery*. 2020;54:285-299. doi:https://dx.doi.org/10.1016/j.yasu.2020.05.006

68. Stucke RS, Sorensen M, Rosser A, Sullivan S. The surgical consult entrustable professional activity (EPA): Defining competence as a basis for evaluation. *American Journal of Surgery*. 2020;219(2):253-257. doi:https://dx.doi.org/10.1016/j.amjsurg.2018.12.056

69. Tanner FC, Brooks N, Fox KF, et al. ESC core curriculumfor the cardiologist. *European Heart Journal*. 2020;41(38):3605-3692. doi:http://dx.doi.org/10.1093/eurheartj/ehaa641

70. Thoma B, Hall AK, Clark K, et al. Evaluation of a National Competency-Based Assessment System in Emergency Medicine: A CanDREAM Study. *Journal of Graduate Medical Education*. 2020;12(4):425-434. doi:https://dx.doi.org/10.4300/JGME-D-19-00803.1

71. Benstead K, Lara PC, Andreopoulos D, et al. Recommended ESTRO Core Curriculum for Radiation Oncology/Radiotherapy 4th edition. *Radiotherapy & Oncology*. 2019;141:1-4. doi:https://dx.doi.org/10.1016/j.radonc.2019.08.013

72. Bonnie LHA, Visser MRM, Bont J, Kramer AWM, van Dijk N. Trainers' and trainees' expectations of entrustable professional activities (EPAs) in a primary care training programme. *Education for Primary Care*. 2019;30(1):13-21. doi:https://dx.doi.org/10.1080/14739879.2018.1532773

73. Brasel KJ, Klingensmith ME, Englander R, et al. Entrustable Professional Activities in General Surgery: Development and Implementation. *Journal of Surgical Education*. 2019;76(5):1174-1186. doi:https://dx.doi.org/10.1016/j.jsurg.2019.04.003

74. Bratberg J. Pharmacy: Addressing substance use in the 21st century. *Substance Abuse*. 2019;40(4):421-434. doi:https://dx.doi.org/10.1080/08897077.2019.1694618

75. Costello L, Argintaru N, Wong A, Simard R, Chacko M, Meshkat N. Addressing unrealistic expectations: A novel transition to discipline curriculum in emergency medicine. *Canadian Journal of Emergency Medicine*. 2019;21(Supplement 1):S48. doi:http://dx.doi.org/10.1017/cem.2019.153

76. Emke AR, Park YS, Srinivasan S, Tekian A. Workplace-Based Assessments Using Pediatric Critical Care Entrustable Professional Activities. *Journal of Graduate Medical Education*. 2019;11(4):430-438. doi:http://dx.doi.org/10.4300/JGME-D-18-01006.1

77. Goodell KH, Ticku S, Fazio SB, Riedy CA. Entrustable Professional Activities in Oral Health for Primary Care Providers Based on a Scoping Review. *Journal of Dental Education*. 2019;83(12):1370-1381. doi:https://dx.doi.org/10.21815/JDE.019.152

78. Greenberg JA, Minter RM. Entrustable Professional Activities: The Future of Competency-based Education in Surgery May Already Be Here. *Annals of Surgery*. 2019;269(3):407-408. doi:http://dx.doi.org/10.1097/SLA.0000000000003153

79. Gubitz G, Poppe AY, Casaubon LK, Shamy M, Khan K, Shoamanesh A. Developing standardized entrustable professional activities (EPAs) for stroke fellowship training in Canada. *International Journal of Stroke*. 2019;14(3 Supplement):44. doi:http://dx.doi.org/10.1177/1747493019872147

80. Hart D, Franzen D, Beeson M, et al. Integration of entrustable professional activities with the milestones for emergency medicine residents. *Western Journal of Emergency Medicine*. 2019;20(1):35-42. doi:http://dx.doi.org/10.5811/westjem.2018.11.38912

81. Johnston L, Sawyer T, Nishisaki A, et al. Neonatal Intubation Competency Assessment Tool: Development and Validation. *Academic pediatrics*. 2019;19(2):157-164. doi:http://dx.doi.org/10.1016/j.acap.2018.07.008

82. Kane SK, Lorant DE. Creation and Validation of Tool to Assess Resident Competence in Neonatal Resuscitation. *Academic pediatrics*. 2019;19(4):394-398. doi:https://dx.doi.org/10.1016/j.acap.2018.09.004

83. Karthikeyan P, Pulimoottil DT. Design and Implementation of Competency Based Postgraduate Medical Education in Otorhinolaryngology: The Pilot Experience in India. *Indian Journal of Otolaryngology & Head & Neck Surgery*. 2019;71(Suppl 1):671-678. doi:https://dx.doi.org/10.1007/s12070-018-1474-5

84. Malter LB, Israel A, Rubin DT. Proposal to Update the Curriculum in Inflammatory Bowel Diseases for Categorical Gastroenterology Fellows. *Inflammatory Bowel Diseases*. 2019;25(9):1443-1449. doi:http://dx.doi.org/10.1093/ibd/izz107

85. Posel N, Hoover ML, Bergman S, Grushka J, Rosenzveig A, Fleiszer D. Objective Assessment of the Entrustable Professional Activity Handover in Undergraduate and Postgraduate Surgical Learners. *Journal of Surgical Education*. 2019;76(5):1258-1266. doi:https://dx.doi.org/10.1016/j.jsurg.2019.03.008

86. Sheth M, Woods RW, Slanetz PJ, Klein K, Fornari A, Lewis P. Development of Breast Imaging Specific Entrustable Professional Activities Using a Double Delphi Technique. *Academic Radiology*. 2019;26(5):591-596. doi:https://dx.doi.org/10.1016/j.acra.2018.10.023

87. Shrivastava SR, Chacko TV, Bhandary S, Shrivastava PS. Development, validation and use of appropriate assessment tools for certification of entrustable professional activities in community medicine to produce a competent postgraduate: A pilot study. *Indian Journal of Public Health*. 2019;63(4):277-281. doi:https://dx.doi.org/10.4103/ijph.IJPH_45_19

88. Smit MP, de Hoog M, Brackel HJL, Ten Cate O, Gemke R. A National Process to Enhance the Validity of Entrustment Decisions for Dutch Pediatric Residents. *Journal of Graduate Medical Education*. 2019;11(4 Suppl):158-164. doi:https://dx.doi.org/10.4300/JGME-D-18-01006

89. Surjadi M, Stringari-Murray S, Saxe JM. Entrustable Professional Activities in Nurse Practitioner Education. *Journal for Nurse Practitioners*. 2019;15(5):e97-e102. doi:10.1016/j.nurpra.2018.12.030

90. Valentine N, Wignes J, Benson J, Clota S, Schuwirth LW. Entrustable professional activities for workplace assessment of general practice trainees. *Medical Journal of Australia*. 2019;210(8):354-359. doi:https://dx.doi.org/10.5694/mja2.50130

91. van Bockel EAP, Walstock PA, van Mook W, et al. Entrustable professional activities (EPAs) for postgraduate competency based intensive care medicine training in the Netherlands: The next step towards excellence in intensive care medicine training. *Journal of Critical Care*. 2019;54:261-267. doi:https://dx.doi.org/10.1016/j.jcrc.2019.09.012

92. Wang ES, Velasquez ST, Smith CJ, et al. Triaging Inpatient Admissions: an Opportunity for Resident Education. *Journal of General Internal Medicine*. 2019;34(5):754-757. doi:http://dx.doi.org/10.1007/s11606-019-04882-2

93. Westein MPD, de Vries H, Floor A, Koster AS, Buurma H. Development of a Postgraduate Community Pharmacist Specialization Program Using CanMEDS Competencies, and Entrustable Professional Activities. *American Journal of Pharmaceutical Education*. 2019;83(6):6863. doi:http://dx.doi.org/10.5688/ajpe6863

94. Aggarwal R. Obstetrics and Gynecology Modified Delphi Survey for Entrustable Professional Activities: Quantification of Importance, Benchmark Levels, and Roles in Simulation-based Training and Assessment. *Cureus*. 2018;10(7)doi:10.7759/cureus.3051

95. Auble B, Saudek K, Hahn D, et al. Entrustable professional activities (EPAS): Mapping performance to milestones. *Academic pediatrics*. 2018;18(5):e6-e7.

96. Barakat LA, Dunne DW, Tetrault JM, et al. The Changing Face of HIV Care: Expanding HIV Training in an Internal Medicine Residency Program. *Academic Medicine*. 2018;93(11):1673-1678. doi:https://dx.doi.org/10.1097/ACM.0000000000002317

97. Borman-Shoap E, Curran D, Naik V, Raushcke T, Ryba-White B. Improving completion rate of real-time EPA-based feedback in the emergency department. *Academic pediatrics*. 2018;18(5):e51-e52.

98. Eckberg K, Borman-Shoap E. Needs assessment and early experience with an outpatient complex care elective. *Academic pediatrics*. 2018;18(5):e28-e29.

99. Emke A, Srinivasan S, Duncan J. Workplace assessment for pediatric critical care fellows using entrustable professional activities. *Critical Care Medicine*. 2018;46(Supplement 1):184. doi:http://dx.doi.org/10.1097/01.ccm.0000528420.71605.ad

100. Hurd C, Back A, Gibbon L, Trowbridge A. Deliberate practice for entrustable professional activities: A point-of care procedural curriculum for delivering serious news and facilitating family conferences in residency training. *Journal of Pain and Symptom Management*. 2018;55(2):634.

101. Ko JJ, Ballard MS, Shenkier T, et al. Validation of a Novel Assessment Tool to Evaluate How Well Medical Residents Are Prepared to Lead Serious Illness Conversations with Oncology Patients, Based on the Competency-Based Medical Education (CBME) Model - A Quantitative Analysis. *Journal of Pain and Symptom Management*. 2018;56(6):e84. doi:http://dx.doi.org/10.1016/j.jpainsymman.2018.10.301

102. Kovatch KJ, Prince MEP, Sandhu G. Weighing Entrustment Decisions with Patient Care during Residency Training. *Otolaryngology - Head & Neck Surgery*. 2018;158(6):1024-1027. doi:https://dx.doi.org/10.1177/0194599818764652

103. McMillan JA, Land ML, Jr., Rodday AM, Wills K, Green CM, Leslie LK. Report of a Joint Association of Pediatric Program Directors-American Board of Pediatrics Workshop: Preparing Future Pediatricians for the Mental Health Crisis. *Journal of Pediatrics*. 2018;201:285-291. doi:https://dx.doi.org/10.1016/j.jpeds.2018.06.044

104. Mink RB, Schwartz A, Herman BE, et al. Validity of Level of Supervision Scales for Assessing Pediatric Fellows on the Common Pediatric Subspecialty Entrustable Professional Activities. *Academic medicine : journal of the Association of American Medical Colleges*. 2018;93(2):283-291. doi:http://dx.doi.org/10.1097/ACM.0000000000001820

105. Pinsk M, Karpinski J, Carlisle E. Introduction of Competence by Design to Canadian Nephrology Postgraduate Training. *Canadian Journal of Kidney Health & Disease*. 2018;5:2054358118786972. doi:https://dx.doi.org/10.1177/2054358118786972

106. Santhosh L, Frank JA. Creation and implementation of a novel entrustable professional activity tool to evaluate fellow ICU rounds leadership and education. *American Journal of Respiratory and Critical Care Medicine Conference: American Thoracic Society International Conference, ATS*. 2018;197(MeetingAbstracts)

107. Schnobrich DJ, Mathews BK, Trappey BE, Muthyala BK, Olson APJ. Entrusting internal medicine residents to use point of care ultrasound: Towards improved assessment and supervision. *Medical Teacher*. 2018;40(11):1130-1135. doi:https://dx.doi.org/10.1080/0142159X.2018.1457210

108. Soran C, Laponis R, Eastburn AP, Thompson V, Summerville S, Julian K. Identifying entrustable professional activities for internal medicine residents in ambulatory continuity practice. *Journal of General Internal Medicine*. 2018;33(2 Supplement 1):713-714.

109. Steiman J, Sullivan SA, Scarborough J, et al. Measuring Competence in Surgical Training through Assessment of Surgical Entrustable Professional Activities. *Journal of Surgical Education*. 2018;75(6):1452-1462. doi:https://dx.doi.org/10.1016/j.jsurg.2018.05.004

110. Taylor DR, Park YS, Smith CA, Karpinski J, Coke W, Tekian A. Creating Entrustable Professional Activities to Assess Internal Medicine Residents in Training: A Mixed-Methods Approach. *Annals of Internal Medicine*. 2018;168(10):724-729. doi:https://dx.doi.org/10.7326/M17-1680

111. Wagner LM, Dolansky MA, Englander R. Entrustable professional activities for quality and patient safety. *Nursing Outlook*. 2018;66(3):237-243. doi:http://dx.doi.org/10.1016/j.outlook.2017.11.001

112. Barratt D, Strasser S. Published Coma Simulation had Face Validity in allowing trainees to demonstrate Neurology Residency Milestones and AAMC Entrustable Professional Activities. *Neurology Conference: 69th American Academy of Neurology Annual Meeting, AAN*. 2017;88(16 Supplement 1)

113. Fehr F, Weis-Becker C, Becker H, Opladen T. Entrustable professional activities in post-licensure training in primary care pediatrics: Necessity, development and implementation of a competency-based post-graduate curriculum. *GMS Journal for Medical Education*. 2017;34(5):Doc67. doi:https://dx.doi.org/10.3205/zma001144

114. Fix OK, Herrine SK, Reddy KG, et al. Entrustable professional activities (EPAS) for transplant hepatology. *Hepatology*. 2017;66(Supplement 1):405A-406A.

115. Kumar HB, Khan AM, Arora VK, Singh N. Fine needle aspiration biopsy: An entrustable professional activity in cytopathology postgraduate training. *Journal of Cytology*. 2017;34(2):84-89. doi:https://dx.doi.org/10.4103/JOC.JOC_67_16

116. Landzaat LH, Barnett MD, Buckholz GT, et al. Development of Entrustable Professional Activities for Hospice and Palliative Medicine Fellowship Training in the United States. *Journal of Pain & Symptom Management*. 2017;54(4):609-616.e1. doi:https://dx.doi.org/10.1016/j.jpainsymman.2017.07.003

117. Mallow M, Baer H, Moroz A, Nguyen VQC. Entrustable Professional Activities For Residency Training in Physical Medicine and Rehabilitation. *American Journal of Physical Medicine & Rehabilitation*. 2017;96(10):762-764. doi:http://dx.doi.org/10.1097/PHM.0000000000000741

118. McMurray L, Hall AK, Rich J, Merchant S, Chaplin T. The Nightmares Course: A Longitudinal, Multidisciplinary, Simulation-Based Curriculum to Train and Assess Resident Competence in Resuscitation. *Journal of Graduate Medical Education*. 2017;9(4):503-508. doi:https://dx.doi.org/10.4300/JGME-D-16-00462.1

119. Moloughney B, Moore K, Dagnone D, Strong D. The development of national entrustable professional activities to inform the training and assessment of public health and preventative medicine residents. *Canadian Medical Education Journal [Electronic Resource]*. 2017;8(3):e71-e80.

120. Moore D, Young CJ, Hong J. Implementing entrustable professional activities: the yellow brick road towards competency-based training? *ANZ Journal of Surgery*. 2017;87(12):1001-1005. doi:http://dx.doi.org/10.1111/ans.14120

121. Parker TA, Guiton G, Jones MD, Jr. Choosing entrustable professional activities for neonatology: a Delphi study. *Journal of Perinatology*. 2017;37(12):1335-1340. doi:https://dx.doi.org/10.1038/jp.2017.144

122. Powell DE, Wallschlaeger A. Making sense of the milestones: entrustable professional activities for pathology. *Human Pathology*. 2017;62:8-12. doi:https://dx.doi.org/10.1016/j.humpath.2016.12.027

123. Srivastava S, Braunlin E, Brown D, et al. Curricula components for entrustable professional activities for the subspecialty of pediatric cardiology. *Progress in Pediatric Cardiology*. 2017;44:17-32. doi:http://dx.doi.org/10.1016/j.ppedcard.2017.01.004

124. Ten Cate O, Tobin S, Stokes ML. Bringing competencies closer to day-to-day clinical work through entrustable professional activities. *Medical Journal of Australia*. 2017;206(1):14-16.

125. Williams JT, Tanverdi M, Okada C. Piloting and assessing an entrustable professional activity (EPA) based evaluation among senior pediatric residents in a denver community hospital. *Academic pediatrics*. 2017;17(5):e58-e59. doi:http://dx.doi.org/10.1016/j.acap.2017.04.159

126. Brown CR, Jr., Criscione-Schreiber L, O'Rourke KS, et al. What Is a Rheumatologist and How Do We Make One? *Arthritis care & research*. 2016;68(8):1166-72. doi:https://dx.doi.org/10.1002/acr.22817

127. Chaplin T, McMurray L, Hall AK. A novel curriculum for assessing competency in resuscitation at the foundations of discipline level of training. *Canadian Journal of Emergency Medicine*. 2016;18(Supplement 1):S63. doi:http://dx.doi.org/10.1017/cem.2016.134

128. Cummings CL. Teaching and assessing ethics in the newborn ICU. *Seminars in Perinatology*. 2016;40(4):261-9. doi:https://dx.doi.org/10.1053/j.semperi.2015.12.016

129. Deitte LA, Gordon LL, Zimmerman RD, et al. Entrustable Professional Activities: Ten Things Radiologists Do. *Academic Radiology*. 2016;23(3):374-81. doi:https://dx.doi.org/10.1016/j.acra.2015.11.010

130. Dwyer T, Wadey V, Archibald D, et al. Cognitive and Psychomotor Entrustable Professional Activities: Can Simulators Help Assess Competency in Trainees? *Clinical Orthopaedics & Related Research®*. 2016;474(4):926-934. doi:10.1007/s11999-015-4553-x

131. Kwan J, Crampton R, Mogensen LL, Weaver R, van der Vleuten CP, Hu WC. Bridging the gap: a five stage approach for developing specialty-specific entrustable professional activities. *BMC Medical Education*. 2016;16:117. doi:https://dx.doi.org/10.1186/s12909-016-0637-x

132. Liu S, O'Riordan J, Rashid P. Exploring a better way to assess urology trainees: Module based whole Entrustable Professional Activities (EPA). *BJU International*. 2016;3):72. doi:http://dx.doi.org/10.1111/bju.13453

133. Manders AJ, Ireland KA, Schumacher DJ, Collier SB, Distler CL, Lenders CM. Development of a nutrition entrustable professional activity for pediatric residents. *FASEB Journal Conference: Experimental Biology*. 2016;30(Meeting Abstracts)

134. Meade LB, Suddarth KH, Jones RR, et al. Patients, Nurses, and Physicians Working Together to Develop a Discharge Entrustable Professional Activity Assessment Tool. *Academic Medicine*. 2016;91(10):1388-1391.

135. Menard-Katcher P, Pigott C, Corral J. The development of a gi motility curriculum using an entrustable professional activity (EPA): A novel process template for creating competency-based medical education driven curricula. *Gastroenterology*. 2016;1):S846.

136. Post JA, Wittich CM, Thomas KG, et al. Rating the Quality of Entrustable Professional Activities: Content Validation and Associations with the Clinical Context. *Journal of General Internal Medicine*. 2016;31(5):518-23. doi:https://dx.doi.org/10.1007/s11606-016-3611-8

137. Touchie C, ten Cate O. The promise, perils, problems and progress of competency-based medical education. *Medical Education*. 2016;50(1):93-100. doi:http://dx.doi.org/10.1111/medu.12839

138. van Houwelingen CT, Moerman AH, Ettema RG, Kort HS, Ten Cate O. Competencies required for nursing telehealth activities: A Delphi-study. *Nurse Education Today*. 2016;39:50-62. doi:https://dx.doi.org/10.1016/j.nedt.2015.12.025

139. Weiss A, Ozdoba A, Carroll V, DeJesus F. Entrustable Professional Activities: Enhancing Meaningful Use of Evaluations and Milestones in a Psychiatry Residency Program. *Academic Psychiatry*. 2016;40(5):850-4. doi:https://dx.doi.org/10.1007/s40596-016-0530-2

140. Wisman-Zwarter N, van der Schaaf M, Ten Cate O, Jonker G, van Klei WA, Hoff RG. Transforming the learning outcomes of anaesthesiology training into entrustable professional activities: A Delphi study. *European Journal of Anaesthesiology*. 2016;33(8):559-67. doi:https://dx.doi.org/10.1097/EJA.0000000000000474

141. Wolfel T, Beltermann E, Lottspeich C, Vietz E, Fischer MR, Schmidmaier R. Medical ward round competence in internal medicine - an interview study towards an interprofessional development of an Entrustable Professional Activity (EPA). *BMC Medical Education*. 2016;16:174. doi:https://dx.doi.org/10.1186/s12909-016-0697-y

142. Adams J, Carroll A. Mixed emotions: A response to - Large MM and Ryan CJ. Suicide risk categorisation of psychiatric inpatients: What it might mean and why it is of no use. Australasian psychiatry 2014; 22(4): 390-2. *Australasian Psychiatry*. 2015;23(1):85-86. doi:http://dx.doi.org/10.1177/1039856214556326

143. Block L, LaVine NA, Verbsky J, Conigliaro J, Chaudhry S. Do internal medicine residents perform patient centered medical home EPAs? A mixed-methods study. *Journal of General Internal Medicine*. 2015;30(22)

144. Clardy PF, Schwartzstein RM. Considering cognition. Current challenges and future directions in pulmonary and critical care fellowship training. *Annals of the American Thoracic Society*. 2015;12(4):474-9. doi:https://dx.doi.org/10.1513/AnnalsATS.201501-054OT

145. Dilly CK. Toward revision of a GI fellowship curriculum to incorporate entrustable professional activities. *Gastroenterology*. 2015;1):S203.

146. Hamburger EK, Lane JL, Agrawal D, et al. The referral and consultation entrustable professional activity: Defining the components in order to develop a curriculum for pediatric residents. *Academic pediatrics*. 2015;15(1):5-8. doi:http://dx.doi.org/10.1016/j.acap.2014.10.012

147. Meade LB, Todd CY, Walsh MM. Found in transition: applying milestones to three unique discharge curricula. *PeerJ*. 2015;3:e819. doi:https://dx.doi.org/10.7717/peerj.819

148. Myers J, Krueger P, Webster F, et al. Development and Validation of a Set of Palliative Medicine Entrustable Professional Activities: Findings from a Mixed Methods Study. *Journal of Palliative Medicine*. 2015;18(8):682-90. doi:https://dx.doi.org/10.1089/jpm.2014.0392

149. Santhosh L, Schaeffer S, Sharpe BA. Honing in on inpatient rounds leadership: Creation & implementation of a noveltoolto evaluate rounds leadership & education. *Journal of General Internal Medicine*. 2015;2):S497.

150. Schultz K, Griffiths J, Lacasse M. The Application of Entrustable Professional Activities to Inform Competency Decisions in a Family Medicine Residency Program. *Academic Medicine*. 2015;90(7):888-97. doi:https://dx.doi.org/10.1097/ACM.0000000000000671

151. Yuan CM, Prince LK, Oliver JD, Abbott KC, Nee R. Implementation of nephrology subspecialty curricular milestones. *American Journal of Kidney Diseases*. 2015;66(1):15-22. doi:http://dx.doi.org/10.1053/j.ajkd.2015.01.020

152. Beeson MS, Kobe D, Bradford A, Warrington SJ. Entrustable professional activities (EPAS)-a framework for the EM milestones. *Academic Emergency Medicine*. 2014;1):S344-S345. doi:http://dx.doi.org/10.1111/acem.12365

153. Beeson MS, Warrington S, Bradford-Saffles A, Hart D. Entrustable professional activities: making sense of the emergency medicine milestones. *Journal of Emergency Medicine*. 2014;47(4):441-52. doi:https://dx.doi.org/10.1016/j.jemermed.2014.06.014

154. Chan B, Englander H, Kent K, et al. Transitioning Toward Competency: A Resident-Faculty Collaborative Approach to Developing a Transitions of Care EPA in an Internal Medicine Residency Program. *Journal of Graduate Medical Education*. 2014;6(4):760-4. doi:https://dx.doi.org/10.4300/JGME-D-13-00414.1

155. Klein MD, Schumacher DJ, Sandel M. Assessing and managing the social determinants of health: defining an entrustable professional activity to assess residents' ability to meet societal needs. *Academic pediatrics*. 2014;14(1):10-3. doi:https://dx.doi.org/10.1016/j.acap.2013.11.001

156. Leipzig RM, Sauvigne K, Granville LJ, et al. What is a geriatrician? American geriatrics society and association of directors of geriatric academic programs end-of-training entrustable professional activities for geriatric medicine. *Journal of the American Geriatrics Society*. 2014;62(5):924-929. doi:http://dx.doi.org/10.1111/jgs.12825

157. Ng LB, Ng Joo Ming M. Entrustable professional activities to enhance continuity of care. *Medical Education*. 2014;48(11):1115. doi:https://dx.doi.org/10.1111/medu.12573

158. O'Keeffe M. Clinical competence in developmental-behavioural paediatrics: raising the bar. *Journal of Paediatrics & Child Health*. 2014;50(1):3-10. doi:https://dx.doi.org/10.1111/jpc.12238

159. Oversight Working N, Rose S, Fix OK, et al. Entrustable professional activities for gastroenterology fellowship training. *Gastrointestinal Endoscopy*. 2014;80(1):16-27. doi:https://dx.doi.org/10.1016/j.gie.2014.05.302

160. Rose S, Fix OK, Shah BJ, Jones TN, Szyjkowski RD. Entrustable professional activities for gastroenterology fellowship training. *Gastroenterology*. 2014;147(1):233-242. doi:http://dx.doi.org/10.1053/j.gastro.2014.04.038

161. Shaughnessy AF, Chang KT, Sparks J, Cohen-Osher M, Gravel J, Jr. Assessing and Documenting the Cognitive Performance of Family Medicine Residents Practicing Outpatient Medicine. *Journal of Graduate Medical Education*. 2014;6(3):526-31. doi:https://dx.doi.org/10.4300/JGME-D-13-00341.1

162. Touchie C, De Champlain A, Pugh D, Downing S, Bordage G. Supervising incoming first-year residents: faculty expectations versus residents' experiences. *Medical Education*. 2014;48(9):921-9. doi:https://dx.doi.org/10.1111/medu.12503

163. Trevallion I, Dhamarrandji C, Dhamarrandji J, et al. How to attain australian indigenous mental health competence: The new 'entrustable professional activities'. *Australian and New Zealand Journal of Psychiatry*. 2014;1):8. doi:http://dx.doi.org/10.1177/0004867414529061

164. van Loon KA, Driessen EW, Teunissen PW, Scheele F. Experiences with EPAs, potential benefits and pitfalls. *Medical Teacher*. 2014;36(8):698-702. doi:https://dx.doi.org/10.3109/0142159X.2014.909588

165. Yuan CM, Prince LK, Zwettler AJ, Nee R, Oliver JD, 3rd, Abbott KC. Assessing achievement in nephrology training: using clinic chart audits to quantitatively screen competency. *American Journal of Kidney Diseases*. 2014;64(5):737-43. doi:https://dx.doi.org/10.1053/j.ajkd.2014.06.027

166. Berberat PO, Harendza S, Kadmon M, Gesellschaft fur Medizinische Ausbildung GMAAfW. Entrustable professional activities - visualization of competencies in postgraduate training. Position paper of the Committee on Postgraduate Medical Training of the German Society for Medical Education (GMA). *GMS Zeitschrift Fur Medizinische Ausbildung*. 2013;30(4):Doc47. doi:https://dx.doi.org/10.3205/zma000890

167. Chang A, Bowen JL, Buranosky RA, et al. Transforming primary care training--patient-centered medical home entrustable professional activities for internal medicine residents. *Journal of General Internal Medicine*. 2013;28(6):801-9. doi:https://dx.doi.org/10.1007/s11606-012-2193-3

168. Hauer KE, Kohlwes J, Cornett P, et al. Identifying entrustable professional activities in internal medicine training. *Journal of Graduate Medical Education*. 2013;5(1):54-9. doi:https://dx.doi.org/10.4300/JGME-D-12-00060.1

169. Hauer KE, Soni K, Cornett P, et al. Developing entrustable professional activities as the basis for assessment of competence in an internal medicine residency: A feasibility study. *Journal of General Internal Medicine*. 2013;28(8):1110-1114. doi:http://dx.doi.org/10.1007/s11606-013-2372-x

170. Shaughnessy AF, Sparks J, Cohen-Osher M, Goodell KH, Sawin GL, Gravel J, Jr. Entrustable professional activities in family medicine. *Journal of Graduate Medical Education*. 2013;5(1):112-8. doi:https://dx.doi.org/10.4300/JGME-D-12-00034.1

171. ten Cate O, Young JQ. The patient handover as an entrustable professional activity: adding meaning in teaching and practice. *BMJ Quality & Safety*. 2012;21 Suppl 1:i9-12. doi:https://dx.doi.org/10.1136/bmjqs-2012-001213

172. Boyce P, Spratt C, Davies M, McEvoy P. Using entrustable professional activities to guide curriculum development in psychiatry training. *BMC Medical Education*. 2011;11:96. doi:https://dx.doi.org/10.1186/1472-6920-11-96

173. ten Cate O, Scheele F. Competency-based postgraduate training: can we bridge the gap between theory and clinical practice? *Academic Medicine*. 2007;82(6):542-7.
